# *Lactobacillus johnsonii‐*FM1 modulates gut microbiota and secretes anticancer metabolite vanillic acid to inhibit colorectal tumorigenesis

**DOI:** 10.1002/imo2.70050

**Published:** 2025-09-03

**Authors:** Wei Lyu, Lu Chen, De‐Feng Li, Shu‐Ying Li, Qian Dai, Hong‐Li Zhou, Yan‐Yan Liu, Jian‐Yun Zhou, Xin‐Jun Liang, Ling Wang

**Affiliations:** ^1^ Clinical Medical Research Center The Second Affiliated Hospital of Army Military Medical University Chongqing China; ^2^ Department of Pharmaceutical Chemistry University of California‐San Francisco San Francisco California USA; ^3^ College of Life Science and Technology, College of Biomedicine and Health Huazhong Agricultural University Wuhan China; ^4^ Institute of Food and Nutrition Development Ministry of Agriculture and Rural Affairs Beijing China; ^5^ Department of Nephrology, Tongji Hospital of Tongji Medical College Huazhong University of Science and Technology Wuhan China; ^6^ Department of Medical Oncology, Hubei Cancer Hospital, Tongji Medical College Huazhong University of Science and Technology Wuhan China

**Keywords:** colorectal cancer, gut microbiota, *Lactobacillus johnsonii*‐FM1, vanillic acid

## Abstract

During colorectal cancer (CRC) progression, probiotics support gut microbial balance, enhance intestinal barrier integrity, and exert antioxidant and anti‐inflammatory effects. Such supplementation may slow tumor growth and serve as an adjunctive therapy for CRC. In this study, we evaluated the impact of *Lactobacillus johnsonii*‐FM1 in *Apc* ^*Min/+*^ and azoxymethane/dextran sulfate sodium‐induced CRC mouse models. Our results demonstrate that *L. johnsonii*‐FM1 markedly reduces tumor number, size, and volume in both models. Shotgun metagenomic sequencing showed that *L. johnsonii*‐FM1 increases the abundance of potentially beneficial taxa while decreasing opportunistic pathogens, thereby preserving gut barrier function. Moreover, untargeted metabolomics paired with liquid chromatography‐tandem mass spectrometry identified vanillic acid (VCA) as a key bioactive metabolite produced by *L. johnsonii*‐FM1. In vitro, VCA inhibits CRC cell line proliferation, diminishes colony formation, induces cell‐cycle arrest, and promotes apoptosis. Mechanistically, VCA attenuates CRC progression by suppressing Wnt/β‐catenin signaling. Our findings suggest a promising probiotic‐based adjunctive strategy for CRC prevention and treatment.

## Highlights

- *Lactobacillus johnsonii*‐FM1 restores gut microbial diversity, enriches beneficial commensals, and depletes potential pathogens in both genetic (*Apc* ^*Min/+*^) and chemical (azoxymethane/dextran sulfate sodium) colorectal cancer models.
- *L. johnsonii*‐FM1 enhances intestinal barrier integrity by upregulating tight junction proteins (ZO‐1, Occludin) and reducing systemic inflammation.
- Metabolomic profiling identifies vanillic acid (VCA) as a key secreted metabolite of *L. johnsonii*‐FM1, produced via a conserved vanillin dehydrogenase enzyme.
- VCA selectively inhibits colorectal cancer cell proliferation, induces cell‐cycle arrest and apoptosis in vitro, and suppresses tumor burden in vivo by attenuating Wnt/β‐catenin signaling.

## Graphical abstract

*Lactobacillus johnsonii*‐FM1 alleviates colorectal tumorigenesis in both *Apc* ^*Min/+*^ and azoxymethane/dextran sulfate sodium mouse models by enriching beneficial gut microbiota, restoring epithelial barrier integrity, suppressing cancer cell proliferation, and promoting apoptosis. Additionally, it produces the antitumor metabolite vanillic acid. Mechanistically, these effects are primarily mediated through inhibition of aberrant activation of the Wnt/β‐catenin signaling pathway.

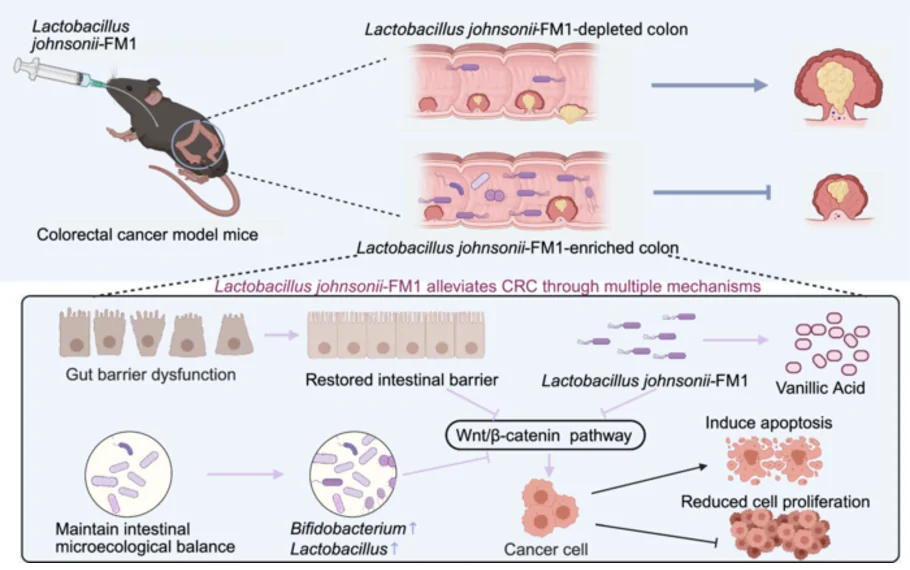

## 1 INTRODUCTION

Colorectal cancer (CRC) is one of the most common malignancies of the digestive tract and ranks fourth in cancer‐related deaths worldwide. Most CRCs develop over 5–10 years through a well‐characterized sequence‐benign adenomatous polyps progress to dysplasia and ultimately to invasive carcinoma‐highlighting the critical window for early intervention [1]. Chemoprevention, involving the use of specific drugs to prevent cancer development, has emerged as a widely utilized and cost‐effective strategy [2]. For instance, nonsteroidal anti‐inflammatory drugs and cyclooxygenase‐2 inhibitors have shown efficacy in reducing CRC incidence and its precancerous lesions in high‐risk populations [3]. However, long‐term use of these medications is associated with a heightened risk of cardiovascular events. Consequently, there is an urgent need to develop novel CRC prevention strategies that minimize adverse effects.

Probiotics have garnered attention for their potential protective effects against CRC by reducing intestinal inflammation, repairing the intestinal barrier, and inhibiting tumor development [4, 5]. Specific probiotic strains, such as *Streptococcus thermophilus* and *Lactobacillus gallinarum*, have demonstrated anticarcinogenic properties [6, 7]. Most Lactobacillus species, classified as lactic acid bacteria (LAB), are commonly found in fermented foods and are recognized for their probiotic potential in humans [8]. The beneficial effects of LAB on various diseases have been extensively documented, and preclinical studies have demonstrated their ability to alleviate chronic inflammation associated with cancer development [6, 8, 9]. *Lactobacillus johnsonii*, a member of the LAB family, has been implicated in a wide range of gastrointestinal and systemic diseases. Previous studies have demonstrated that *L. johnsonii* can ameliorate *Helicobacter pylori* infection [10, 11], colitis [12, 13], *Escherichia coli*‐induced diarrhea [14, 15]. Furthermore, it has been reported to play protective roles in conditions such as respiratory infections [16, 17], diabetes [18, 19], liver diseases [20, 21], arthritis [22, 23], memory impairment [24], and lung injury [25, 26]. These findings highlight its broad immunomodulatory and anti‐inflammatory potential. Consistently, *L. johnsonii* abundance is markedly reduced in mouse models of CRC induced by *Apc* ^*Min/+*^ or azoxymethane (AOM)/dextran sodium sulfate (DSS) [27]. Similarly, human CRC tissues exhibit significantly lower levels of *L. johnsonii* compared to adjacent normal tissues [28]. These findings suggest that *L. johnsonii* may play a role in suppressing CRC development. In this study, we investigate the ability of *L. johnsonii*‐FM1 to suppress colorectal tumorigenesis in both mouse models and CRC cell lines through the promotion of apoptosis. Our findings indicate that this tumor‐suppressive effect is mediated by vanillic acid (VCA), a metabolite produced by *L. johnsonii*‐FM1.

## 2 RESULTS

### *Lactobacillus johnsonii*‐FM1 protects against colorectal cancer in *Apc* ^*Min/+*^ mice and AOM/DSS‐treated mice

**FIGURE 1:**
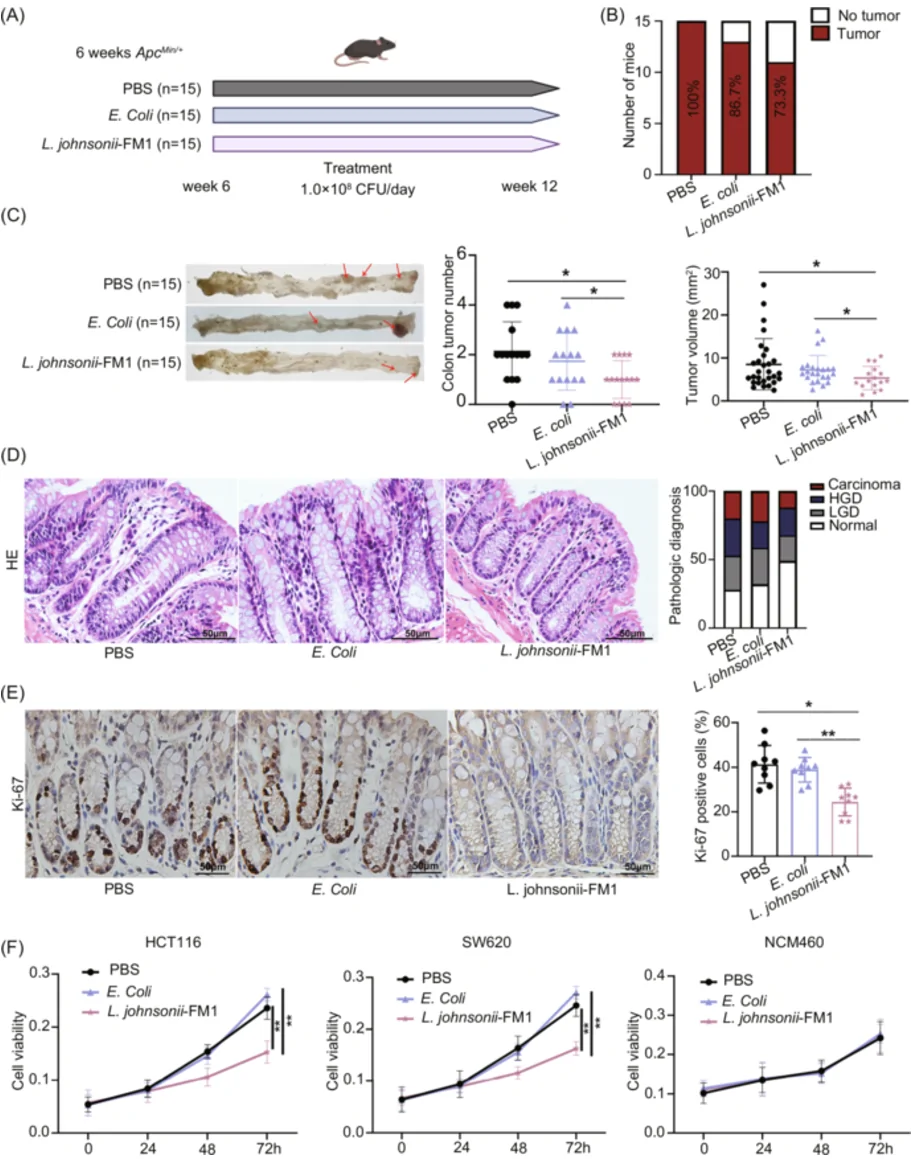
*Lactobacillus johnsonii*‐FM1 inhibits gut tumorigenesis in *Apc* ^*Min/+*^ mice. (A) Schematic diagram illustrating the experimental design and timeline for the *Apc* ^*Min/+*^ mouse model. (B) The number of mice with hematochezia events was recorded for each group, and the percentage of affected mice was calculated (*n* = 15). (C) Representative images of colon tumors from the *Apc* ^*Min/+*^ mouse model. The total number of tumors and their sizes were quantified across different treatment groups. Red arrows indicate visible colon tumors. (D) Hematoxylin and eosin (H&E) staining was used for pathological evaluation of the mouse colons. Pathological scores were assigned as follows: 0 for normal, 1 for low‐grade dysplasia (LGD), 2 for high‐grade dysplasia (HGD), and 3 for carcinoma, with quantitative analysis provided. (E) Immunohistochemical (IHC) staining for Ki‐67 in mouse colons. Quantitative analysis of the Ki‐67 index was performed to assess proliferative activity. (F) Cell growth curves of CRC cell lines (HCT116, SW620) and normal epithelial cell line (NCM460) treated with *L. johnsonii*‐FM1, *E. coli*, and PBS. The Y‐axis shows optical density at 570 nm (OD₅₇₀), which corresponds to MTT formazan absorbance and is proportional to the number of viable cells. Data are expressed as mean ± Standard Deviation (SD). Statistical significance was determined by 1‐way or 2‐way analysis of variance, where appropriate. ∗∗*p* < 0.01, ∗*p* < 0.05. Dot plots reflect data points from independent experiments.

To investigate the effect of *L. johnsonii*‐FM1 on colorectal tumorigenesis, *Apc* ^*Min/+*^ mice were gavaged once daily for 8 weeks with *L. johnsonii*‐FM1 (1.0 × 10^8^ CFU), non‐tumorigenic *E. coli* MG1655 (1.0 × 10^8^ CFU) as a bacterial control, or phosphate‐buffered saline (PBS) (Figure 1A). After 8 weeks of treatment, mice receiving *L. johnsonii*‐FM1 exhibited a lower incidence of bloody stool compared with the PBS‐treated group (Figure 1B). Post‐mortem analysis revealed a significant reduction in both the number and volume of tumors in mice treated with *L. johnsonii*‐FM1 compared to controls (Figure 1C). Histological examination of colon samples revealed that mice treated with *L. johnsonii*‐FM1 had a significantly lower incidence of adenocarcinoma and both high‐ and low‐grade dysplasia than control mice (Figure 1D). Additionally, *L. johnsonii*‐FM1 treatment mice showed a notable reduction in Ki‐67‐positive cells within the colon sections, suggesting decreased cellular proliferation (Figure 1E). Next, we determined whether *L. johnsonii*‐FM1 had a direct antitumorigenic effect in vitro. Colon cancer cell lines HCT116 and SW620, and colon normal epithelial cell line NCM460 were cocultured with or without *L. johnsonii*‐FM1. *E. coli* and PBS were used as bacterial and blank controls, respectively. Coculture with *L. johnsonii*‐FM1 resulted in a significant decrease in the viability of colon cancer cell lines (HCT116 and SW620), while having no detrimental effect on the viability of normal epithelial cells (NCM460) (Figure 1F). These results indicated that *L. johnsonii*‐FM1 preferentially inhibited CRC cell viability.

During the period of gavage, there was no difference in body weight among groups (Figure S1A). Critically, Histological analysis of liver and kidney by H&E revealed no abnormalities (Figure S1B). Moreover, *L. johnsonii*‐FM1 did not alter serum alanine transaminase, aspartate transaminase (liver function), and blood urea nitrogen and creatinine (kidney function) (Figure S1C–F), indicating that *L. johnsonii*‐FM1 is safe for use in mouse models, with no detectable organ toxicity.

To further substantiate the chemopreventive potential of *L. johnsonii*‐FM1, we assessed its effects in an AOM/DSS‐induced colorectal cancer model (Figure S2A). After 8 weeks of treatment, mice in the *L. johnsonii*‐FM1 group exhibited a significantly reduced incidence of bloody stools relative to the control group (Figure S2B). These observations were corroborated by a marked decrease in both the number and size of colon tumors in the *L. johnsonii*‐FM1‐treated mice (Figure S2C). Furthermore, *L. johnsonii*‐FM1 decreased the incidence of colonic carcinoma (Figure S2D) and reduced Ki‐67‐positive cell frequency (Figure S2E). These findings underscored the chemopreventive efficacy of *L. johnsonii*‐FM1 in both carcinogen‐induced colorectal cancer models and the *Apc* ^*Min/+*^ transgenic mouse model.

### *Lactobacillus johnsonii*‐FM1 modulates the gut microbiota of *Apc* ^*Min/+*^ mice

**FIGURE 2:**
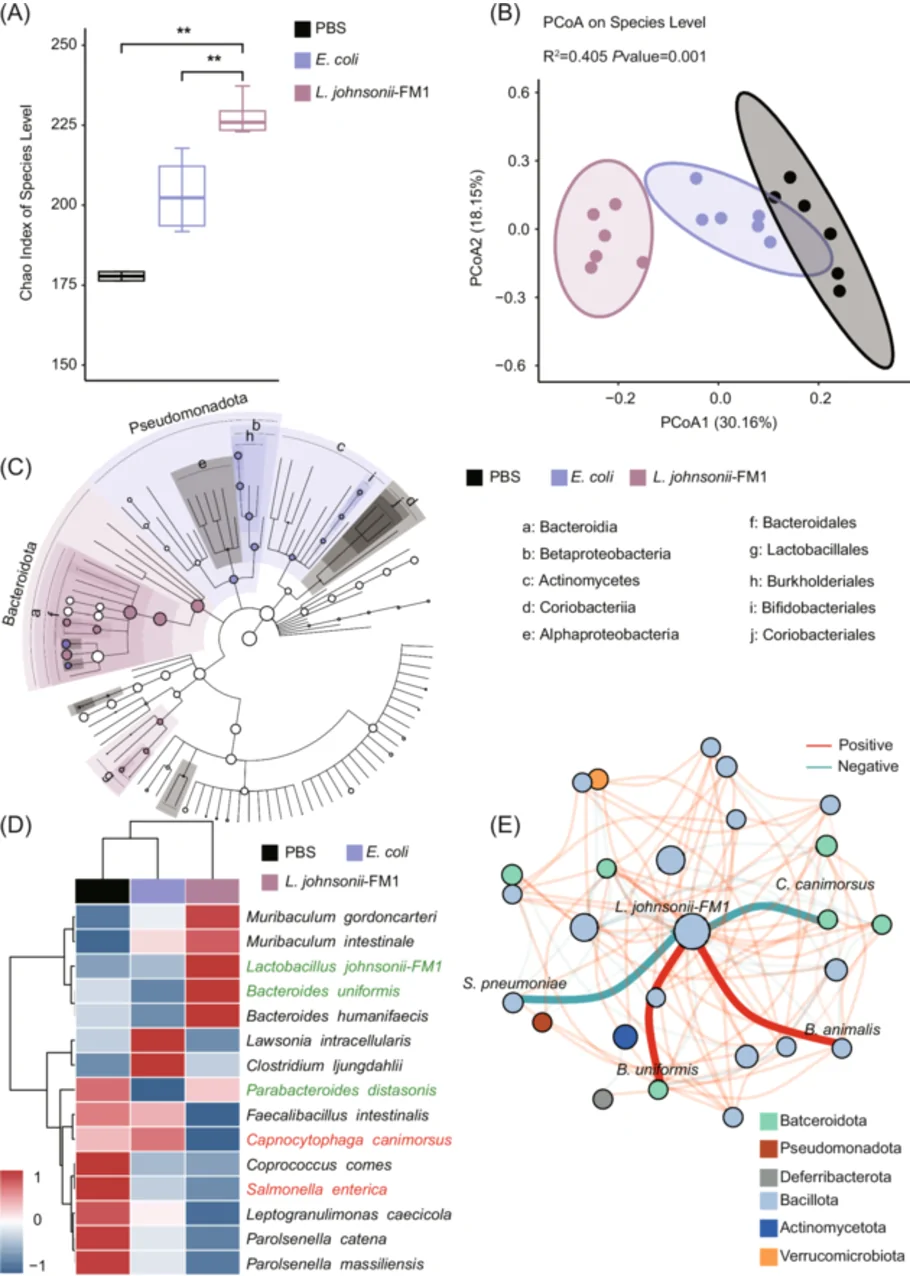
*Lactobacillus johnsonii*‐FM1 alters gut microbial composition and increases the abundance of potential probiotics in the *Apc* ^*Min/+*^ model mice. (A, B) Chao Index (α‐diversity) and PCoA2 analysis (β‐diversity) of the gut microbiota in control and *L. johnsonii*‐FM1‐treated mice from the *Apc* ^*Min/+*^ model. Comparisons of α‐ and β‐diversity were accessed by two‐sided Mann–Whitney *U* test and permutational multivariate analysis of variance, respectively. (C) Linear discriminant analysis Effect Size ring tree diagram showing microbial distribution among the three groups. (D) Identification of marker microbes differentiating groups between *L. johnsonii*‐FM1 and *E. Coli* or between the PBS (*p* < 0.05, LDA > 2). (E) Co‐occurrence analysis: Spearman correlation coefficients between microbes. Different colors represent different bacteria. Red lines indicate positive correlations, blue lines indicate negative correlations, and dark colored lines indicate no correlation. Dot plots reflect data points from independent experiments.

Probiotics were reported to increase gut microbiota diversity, promote the growth of beneficial bacteria, and maintain gut microbiota balance, thereby alleviating the progression of colorectal cancer development [6]. To investigate whether *L. johnsonii*‐FM1 modulates gut microbiota during colorectal tumorigenesis, we conducted shotgun metagenomic sequencing on fecal samples from *Apc* ^*Min/+*^ models treated with *L. johnsonii*‐FM1, *E. coli*, or PBS. At baseline, no significant differences in α‐ or β‐diversity were observed between groups. However, *L. johnsonii*‐FM1 treatment significantly increased α‐diversity and microbial abundance compared to PBS or *E. coli* groups (Figure 2A). Principal coordinate analysis of β‐diversity analysis revealed distinct alterations in gut microbiota composition in the *L. johnsonii*‐FM1 group (Figure 2B). Consistent with the above results, the tree branching diagram showed that the composition of the gut microbiota was different among the various groups (Figure 2C). Together, these data indicated that *L. johnsonii*‐FM1 could reshape the gut microbiota composition during colorectal tumorigenesis.

Although no significant differences were observed at the phylum level between the control and *L. johnsonii*‐FM1 group (Figure S3A,B), at the species level were significantly affected by the *L. johnsonii*‐FM1 treatment in *Apc* ^*min/+*^ models. Potentially beneficial commensals *Bacteroides uniformis* and *Bacteroides animalis* were enriched by *L. johnsonii*‐FM1 treatment, together with depletion of potentially pathogenic bacteria *Capnocytophaga canimorsus* and *Salmonella enterica* (Figure 2D and Table S1). Next, we constructed ecological networks from differential bacteria identified between *L. johnsonii*‐FM1 and control groups (Figure 2E). Co‐exclusive correlations were found between potentially probiotics and potentially pathogenic bacteria in *L. johnsonii*‐FM1‐treated mice, including *L. johnsonii*‐FM1–*Bacteroides uniformis*, *L. johnsonii*‐FM1‐*Bacteroides animalis*, *L. johnsonii*‐FM1‐*Capnocytophaga canimorsus*, and *L. johnsonii*‐FM1‐*Salmonella enterica* implying that *L. johnsonii*‐FM1‐enriched potentially probiotics might antagonize pathogenic bacteria in CRC (Figure 2E). Taken together, these data suggested that *L. johnsonii*‐FM1 might reverse microbial dysbiosis in CRC.

In parallel, we also profiled the fecal microbiome of AOM/DSS‐treated mice following daily gavage with *L. johnsonii*‐FM1, *E. coli* or PBS. As in the *Apc* ^*Min/+*^ model, mice treated with *L. johnsonii*‐FM1 markedly increased α‐diversity (Figure S4A) and shifted overall community structure by β‐diversity compared with controls (Figure S4B). A heatmap of species‐level abundances highlighted that *L. johnsonii*‐FM1 gavage enriched the potentially beneficial (*Ligilactobacillus murinus* [29]) and depleted potential pathogens (*Klebsiella pneumonia* [30], *Streptococcus gallolyticus* [31]) (Figure S4C). These results demonstrated that *L. johnsonii*‐FM1 consistently restored microbial diversity, enriched beneficial taxa, and suppressed opportunistic pathogens in both genetic and chemically induced CRC models.

### *Lactobacillus johnsonii*‐FM1 restores gut barrier function in colorectal cancer mice

**FIGURE 3:**
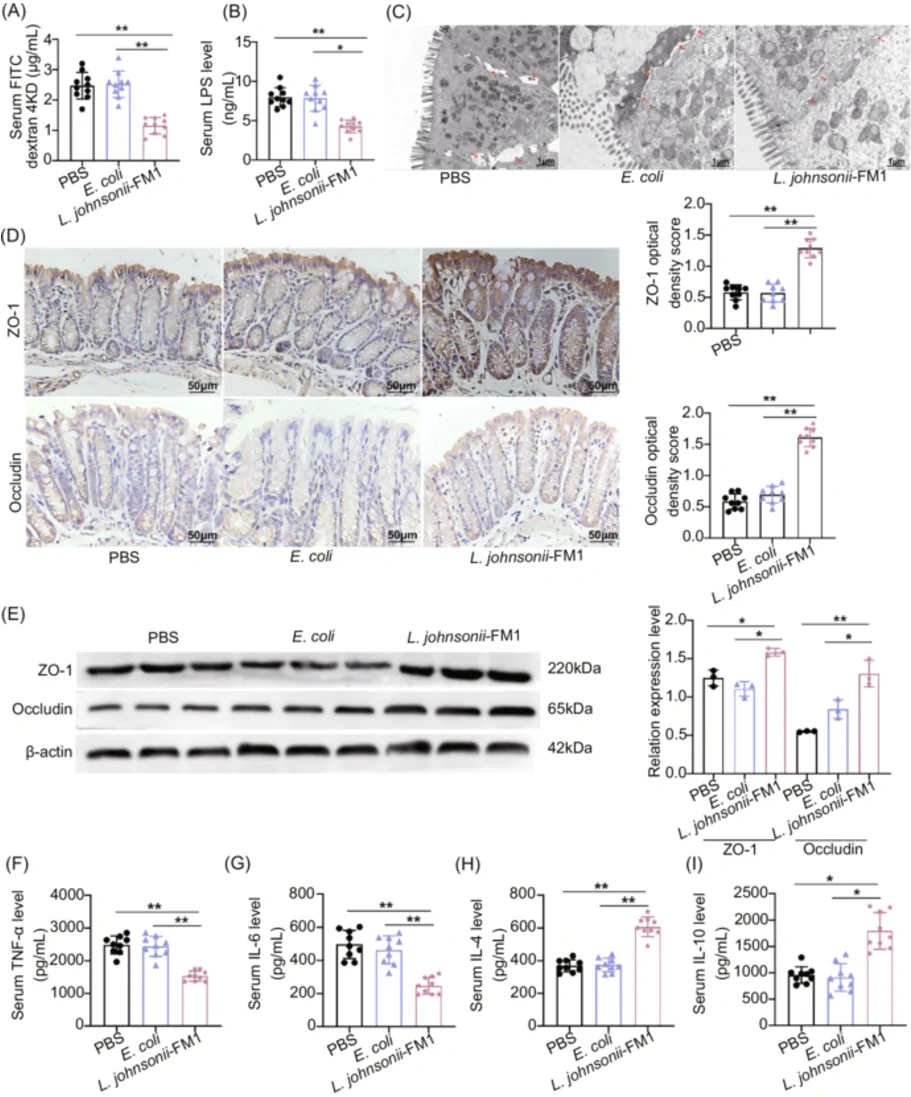
*Lactobacillus johnsonii*‐FM1 restores CRC‐induced gut barrier dysfunction. (A) Serum FITC‐dextran 4 KD concentration and (B) lipopolysaccharide (LPS) concentration of control and *L. johnsonii*‐FM1‐treated mice from *Apc* ^Min/+^ model (*n* = 9). (C) Representative images of intercellular junctions of control and *L. johnsonii*‐treated mice by transmission electron microscopy (TEM). (D) Immunohistochemical (IHC) for distribution of the adhesion molecule ZO‐1 and Occludin with quantitative analysis in colon tissues of PBS, *Ecoli*, and *L. johnsonii*‐FM1‐treated mice from *Apc* ^Min/+^ model. (E) Expression of gut barrier–associated proteins ZO‐1 and Occludin in colon tissues of PBS, *E. coli* and *L. johnsonii*‐FM1‐treated mice from *Apc* ^Min/+^ model by Western blot. (F, G) Pro‐inflammatory TNF‐α and interleukin‐6 (IL‐6) concentrations and (H, I) anti‐inflammatory IL‐4 and IL‐10 concentrations in serum of *L. johnsonii*‐FM1‐treated group and control group in an *Apc* ^Min/+^ model. Data are expressed as mean ± SD. Statistical significance was determined by 1‐way or 2‐way analysis of variance, where appropriate. ∗∗*p* < 0.01, ∗*p* < 0.05. Dot plots reflect data points from independent experiments.

Gut dysbiosis was reported to play a critical role in compromising gut barrier integrity. To determine whether *L. johnsonii*‐FM1 improved gut barrier function, we performed fluorescein isothiocyanate (FITC)‐dextran intestinal permeability assays in *Apc* ^*Min/+*^ mice. Results showed that *L. johnsonii*‐FM1‐treated mice exhibited lower serum FITC–dextran and serum bacterial lipopolysaccharide levels (Figure 3A‐B), indicating reduced gut permeability. Additionally, transmission electron microscopy revealed partial restoration of gut barrier structure (Figure 3C). Immunohistochemical analysis showed enhanced expression of tight junction proteins, ZO‐1 (a key component of tight junctions and Occludin (a cell adhesion molecule), which serves as a marker of gut barrier integrity, in the *L. johnsonii*‐FM1‐treated group (Figure 3D), corroborated by Western blot analysis (Figure 3E). Given that increased gut permeability could contribute to chronic inflammation [32], we further assessed the inflammatory status of *Apc* ^*Min/+*^ mice. ELISA results indicated that *L. johnsonii*‐FM1 significantly reduced the expression of pro‐inflammatory cytokines, including tumor necrosis factor‐α (TNF‐α) and interleukin‐6 (IL‐6), while enhancing the levels of anti‐inflammatory cytokines interleukin‐4 (IL‐4) and interleukin‐10 (IL‐10) (Figure 3F–I). Together, these data indicated that *L. johnsonii*‐FM1 reinforced gut barrier integrity and reduced systemic inflammation in *Apc* ^*Min/+*^ mice.

To corroborate these findings, we employed the AOM/DSS‐treated C57BL/6 mouse model to ascertain whether the effect of *L. Johnsonii*‐FM1 was specific to a particular CRC mouse model. Analogous to the *Apc* ^*Min/+*^ model, *L. johnsonii*‐FM1 enhanced gut barrier function and reduced inflammation levels in AOM/DSS‐treated mice (Figure S5A–I). These consistent findings suggested that *L. johnsonii*‐FM1 suppressed colorectal tumor development across two distinct CRC models, likely by restoration of barrier integrity and mitigation of inflammation to protect against tumorigenesis.

### *Lactobacillus johnsonii*‐FM1 supernatant induces apoptosis and inhibits viability in CRC cells

**FIGURE 4:**
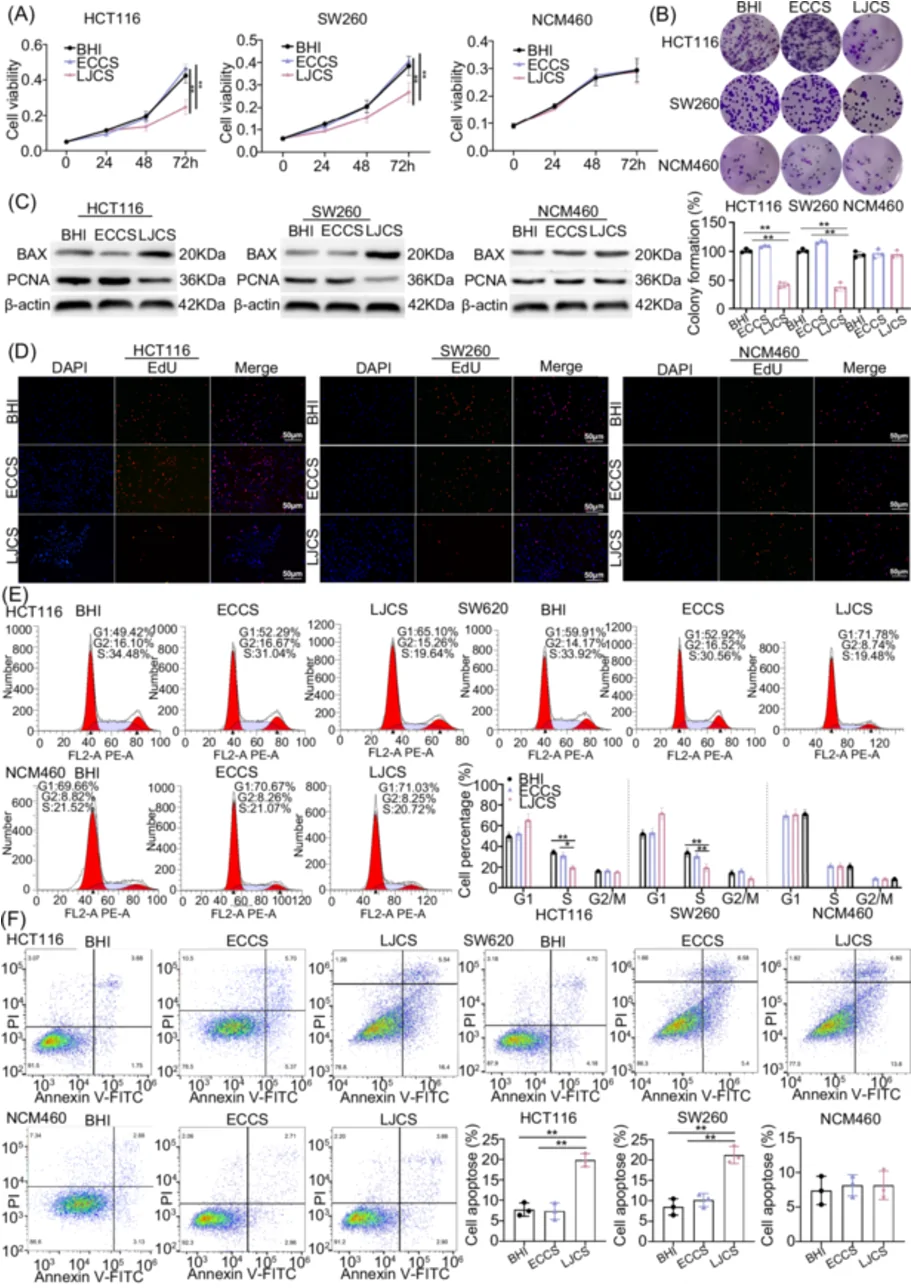
*Lactobacillus johnsonii*‐FM1 supernatant inhibits the viability of colon cancer cells. (A) *Lactobacillus johnsonii*‐FM1 conditioned supernatant (LJCS) reduced the colonic cell viability, except NCM460. *Escherichia coli* conditioned supernatant (ECCS) and brain heart infusion (BHI) were used as control. The *Y*‐axis shows optical density at 570 nm (OD₅₇₀), which corresponds to MTT formazan absorbance and is proportional to the number of viable cells. (B) LJCS suppressed the colony formation of CRC cells. (C) LJCS reduced proliferating cell nuclear antigen (PCNA) and increased Bcl‐2‐associated X protein (BAX) levels in CRC cells. (D) LJCS reduced EdU‐positive cells. (E) LJCS induced CRC cell cycle arrest at Gap 1 (G1) phase/Synthesis (S) phase. (F) LJCS increased CRC cell apoptosis. BHI, the brain heart infusion; ECCS, the culture supernatant of *E. coli*; LJCS, the culture supernatant of *L. johnsonii*‐FM1; PI, Propidium Iodide. Data are expressed as mean ± SD. Statistical significance was determined by one‐way or two‐way analysis of variance, where appropriate. ∗∗*p* < 0.01, ∗*p* < 0.05. Dot plots reflect data points from independent experiments.

To assess the tumor‐suppressive effects of *L. johnsonii*‐FM1 in vitro, we used CRC cell lines HCT116 and SW620 alongside the normal colonic epithelial cell line NCM460. Treatment with *L. johnsonii*‐FM1 culture supernatant (LJCS) significantly inhibited the viability of CRC cells compared to the *E. coli* culture supernatant (ECCS) and brain heart infusion (BHI) control groups, while having no negative impact on normal colonic cells (Figure 4A). The growth‐suppressing effect was further validated by colony formation assays (Figure 4B), decreased proliferating cell nuclear antigen (PCNA) protein levels (Figure 4C), and reduced EdU‐positive cells (Figure 4D). Meanwhile, cell‐cycle analysis showed that LJCS treatment arrested cell cycle progression from Gap 1 (G1) phase to Synthesis (S) phase in HCT116 and SW620 cells (Figure 4E). To elucidate the mechanism by which LJCS inhibited CRC cell viability, we performed quantitative analyses of apoptosis and cell cycle distribution. Our findings revealed that LJCS significantly induced apoptosis in colorectal cancer cell lines (HCT116 and SW620), while leaving normal colonic epithelial cells (NCM460) unaffected (Figure 4F). This proapoptotic effect was further corroborated by the elevated expression of the BAX protein, a crucial regulator of apoptosis (Figure 4C). Together, these data demonstrated that metabolites secreted by *L. johnsonii*‐FM1 effectively suppressed CRC cell viability and colony formation, highlighting their potential as anticancer agents.

### *Lactobacillus johnsonii*‐FM1 alters gut metabolite composition to protect against CRC

**FIGURE 5:**
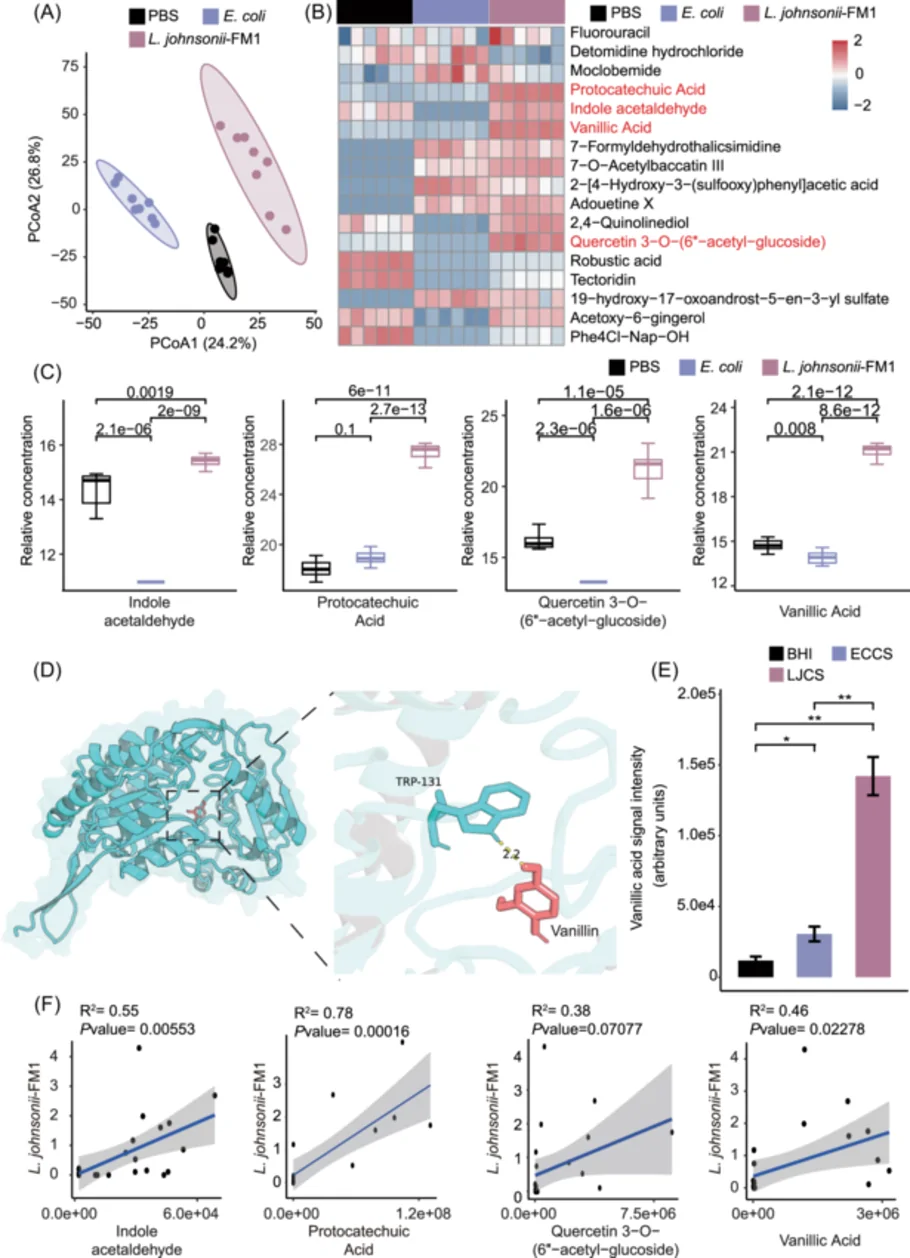
*Lactobacillus johnsonii*‐FM1 alters intestinal metabolite composition. (A) Principal component analysis plot for gut metabolomics analysis in PBS, *E. coli*, and *L. johnsonii*‐FM1. (B) Identification of marker metabolites differentiating PBS, *E. coli*, and *L. johnsonii*‐FM1. (C) Four metabolites of high abundance in *L. johnsonii*‐FM1‐treated *Apc* ^*Min*/+^ mice: Indole acetaldehyde, protocatechuic acid, quercetin 3‐O‐(6”‐acetyl‐glucoside), and vanillic acid. (D) Predicted binding of vanillin to the putative VDH‐like protein in *L. johnsonii*‐FM1. Vanillin (red) docks into a defined pocket of the FM1‐encoded protein, forming a hydrogen bond with residue Trp131 at 2.2 Å. The pocket exhibits typical characteristics of aldehyde dehydrogenases, supporting potential functional relevance. (E) Quantification of vanillic acid signal intensity in BHI, ECCS, and LJCS samples. (F) Correlation analysis of *L. johnsonii*‐FM1 with Indole acetaldehyde, Protocatechuic Acid, Quercetin 3‐O‐(6”‐acetyl‐glucoside) and vanillic acid. Dot plots reflect data points from independent experiments. BHI, the brain heart infusion; ECCS, the culture supernatant of *E. coli;* LJCS, the culture supernatant of L. johnsonii‐FM1.

Given that metabolites secreted by the gut microbiota played an important role in regulating health and disease [33], we sought to identify anti‐CRC molecules produced by *L. johnsonii*‐FM1. Accordingly, we performed untargeted liquid chromatography‐tandem mass spectrometry (LC‐MS/MS) on fecal samples from *Apc* ^*Min/+*^ mice treated with PBS, *E. coli*, or *L. johnsonii*‐FM1. Principal component analysis score plots revealed clear separation among the PBS, *E. coli* and *L. johnsonii*‐FM1 (Figure 5A). Differential abundance analysis identified metabolites uniquely enriched in the *L. johnsonii*‐FM1 group relative to PBS and *E. coli* controls (Figure 5B and Tables S2–4). Among the top upregulated outliers in the *L. johnsonii* FM1‐treated mice were VCA, indole‐3‐acetaldehyde, protocatechuic acid, and Quercetin 3‐O‐(6″‐acetyl‐glucoside) (Figure 5C).

Vanillin dehydrogenase (VDH) catalyzes the NAD⁺‐dependent oxidation of vanillin—whether derived endogenously or from dietary polyphenols—into VCA (4‐hydroxy‐3‐methoxybenzoic acid) [34]. To identify a VDH candidate in *L. johnsonii*, we searched six publicly available *L. johnsonii* genomes for enzymes capable of vanillin oxidation. BLASTP comparison to a characterized VDH sequence, together with Pfam PF00171 domain analysis, revealed a conserved family of aldehyde dehydrogenase‐like proteins (~28% identity, e‐value < 1 × 10^−^⁴⁸). All candidate homologs retained the key active‐site residues and cofactor‐binding motifs characteristic of VDH enzymes (Table S5). To further assess the VDH‐like candidate in *L. johnsonii*‐FM1, we modeled vanillin docking onto both the *L. johnsonii*‐FM1‐encoded protein and a reference VDH with known catalytic activity. The vanillin (highlighted in red) occupies a similarly shaped pocket in each enzyme. In *L. johnsonii*‐FM1's protein, vanillin forms a 2.2 Å hydrogen bond with Trp131, while in the validated VDH it engages Trp148 at 2.0 Å (Figure 5D and Figure S6). The comparable ligand orientations, interaction distances, and conserved pocket topologies indicated a shared substrate‐recognition mechanism. These structural insights reinforced our hypothesis that the *L. johnsonii*‐FM1‐encoded enzyme functioned as a bona fide VDH, catalyzing the conversion of vanillin to VCA.

To further investigate whether VCA is a metabolic product of *L. johnsonii*‐FM1, we conducted untargeted metabolomic analysis comparing LJCS to the medium alone. The results showed that the VCA levels were significantly higher in the *L. johnsonii*‐FM1 supernatant (Figure 5E), confirming VCA as an *L. johnsonii*‐FM1‐derived metabolite and supporting our prediction that *L. johnsonii*‐FM1 encoded a functional VDH enzyme catalyzing the conversion of vanillin to VCA. Next, we classified the sources of these differential metabolites. The results found that, in addition to other types, metabolites from microorganisms, hosts, and shared metabolites accounted for most of the remaining metabolites (Figure S7A). Enrichment analysis of co‐upregulated metabolites from the *L. johnsonii*‐FM1 revealed that the tryptophan metabolism pathway was enhanced (Figure S7B). In addition, the Spearman correlation coefficient showed that the three metabolites were positively correlated with *L. johnsonii*‐FM1 (Figure 5F). We anticipated that *L. johnsonii*‐FM1‐produced VCA could be responsible for the anti‐CRC effect exhibited by *L. johnsonii*‐FM1.

### VCA inhibits CRC development by modulating the wnt/β‐catenin signaling pathway

**FIGURE 6:**
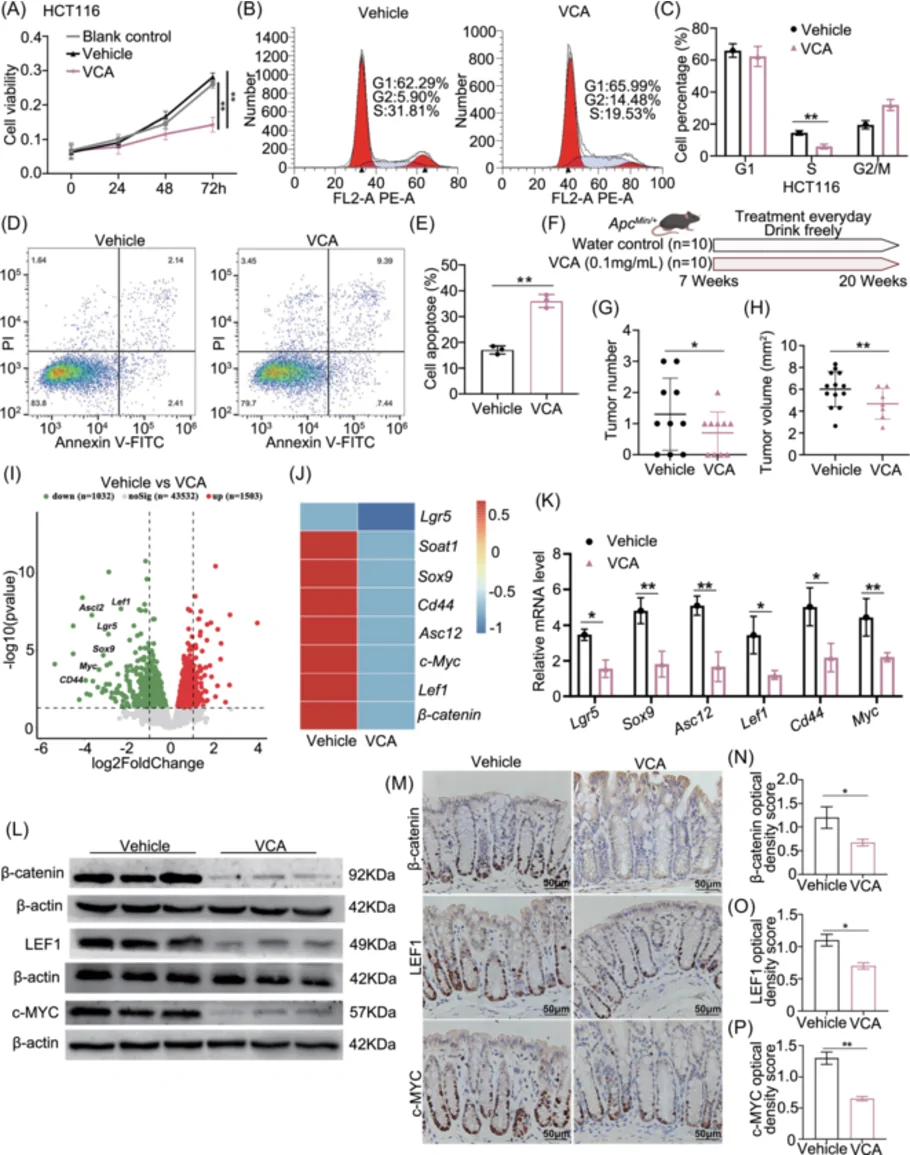
Vanillic acid (VCA) inhibits cell proliferation and cell junction impairment by modulating the Wnt/β‐catenin signaling pathway. (A) VCA reduced the colonic cell viability, except NCM460. The *Y*‐axis shows optical density at 570 nm (OD₅₇₀), which corresponds to MTT formazan absorbance and is proportional to the number of viable cells. (B) VCA induced CRC cell cycle arrest at Synthesis (S) phase. (C) Quantitative analysis of cell cycle treated with VCA. (D, E) Representative images and quantitative analysis of cell apoptosis in CRC cell lines treated with VCA and Vehicle. (F) Schematic diagram showing the experimental design, timeline and representative macroscopic images of colons from of VCA‐treated *Apc* ^Min/+^ mouse model. (G, H) Colon tumor number and size in *Apc* ^Min/+^ mice with or without VCA treatment. (I, J) The differentially expressed genes and heat‐map of RNA expression in mouse colon tissues. (K) RT‐qPCR results of differentially expressed genes in mouse colons. (L) Western blot results of differentially expressed genes in mouse colons. (M–P) Immunohistochemical (IHC) for distribution of the adhesion molecule β‐catenin, LEF1, and c‐MYC with quantitative analysis in colon tissues of vehicle and VCA‐treated mice from *Apc* ^Min/+^ model. Data are expressed as mean ± SD. ∗∗*p* < 0.01, ∗*p* < 0.05. Dot plots reflect data points from independent experiments.

We evaluated the functional effects of VCA in CRC cell growth in vitro and in CRC tumorigenesis in vivo. Coculture tests demonstrated that cell viability of CRC cells (HCT116 and SW620) but not normal colonic epithelial cell NCM460 was substantially reduced when exposed to VCA at a concentration comparable to that of LGCS (Figure 6A and Figure S8A,B). This growth inhibition was further confirmed by reduced EdU incorporation (Figure S8C–E). Meanwhile, cell cycle analysis showed that VCA treatment moderated cell cycle progression in HCT116 and SW620 cells compared with vehicle controls (Figure 6B,C and Figure S8F–I). Moreover, VCA significantly increased apoptosis in CRC cells, with no effect on normal epithelial colonic cells NCM460 cells (Figure 6D,E; Figure S8J–K). For in vivo experiments, gavage of VCA (20 mg/kg) into *Apc* ^*Min/+*^ mice (Figure 6F) significantly reduced tumor number and tumor size (Figure 6G,H). Thus, VCA could contribute, at least in part, to the tumor‐suppressive effect of *L. johnsonii*‐FM1.

To further investigate the potential mechanisms by which VCA antagonized colorectal tumor progression, we conducted RNA sequencing on the colon tissues of VCA‐treated mice and control mice. The resulting volcano plot and heatmap showed that VCA treatment downregulated the expression of stemness‐related and cell‐cycle‐promoting genes, including Leucine‐rich repeat‐containing G‐protein coupled receptor 5 (*Lgr5*), Sex‐determining region Y‐box 9 (*Sox9*), Cluster of Differentiation 44 (*Cd44*), Myelocytomatosis oncogene (*c‐Myc*), Achaete‐Scute Family BHLH Transcription Factor 2 (*Ascl2*), and Lymphoid Enhancer‐Binding Factor 1 (*Lef1*) (Figure 6I,J). These genes were known targets or downstream effectors of the Wnt/β‐catenin pathway. RT‐qPCR confirmed a substantial decrease in the expression of these genes (Figure 6K). Moreover, Western blot and IHC staining demonstrated a significant reduction in β‐catenin, LEF1, and c‐MYC protein expression in colon tissues from VCA‐treated mice (Figure 6L–P).

**FIGURE 7:**
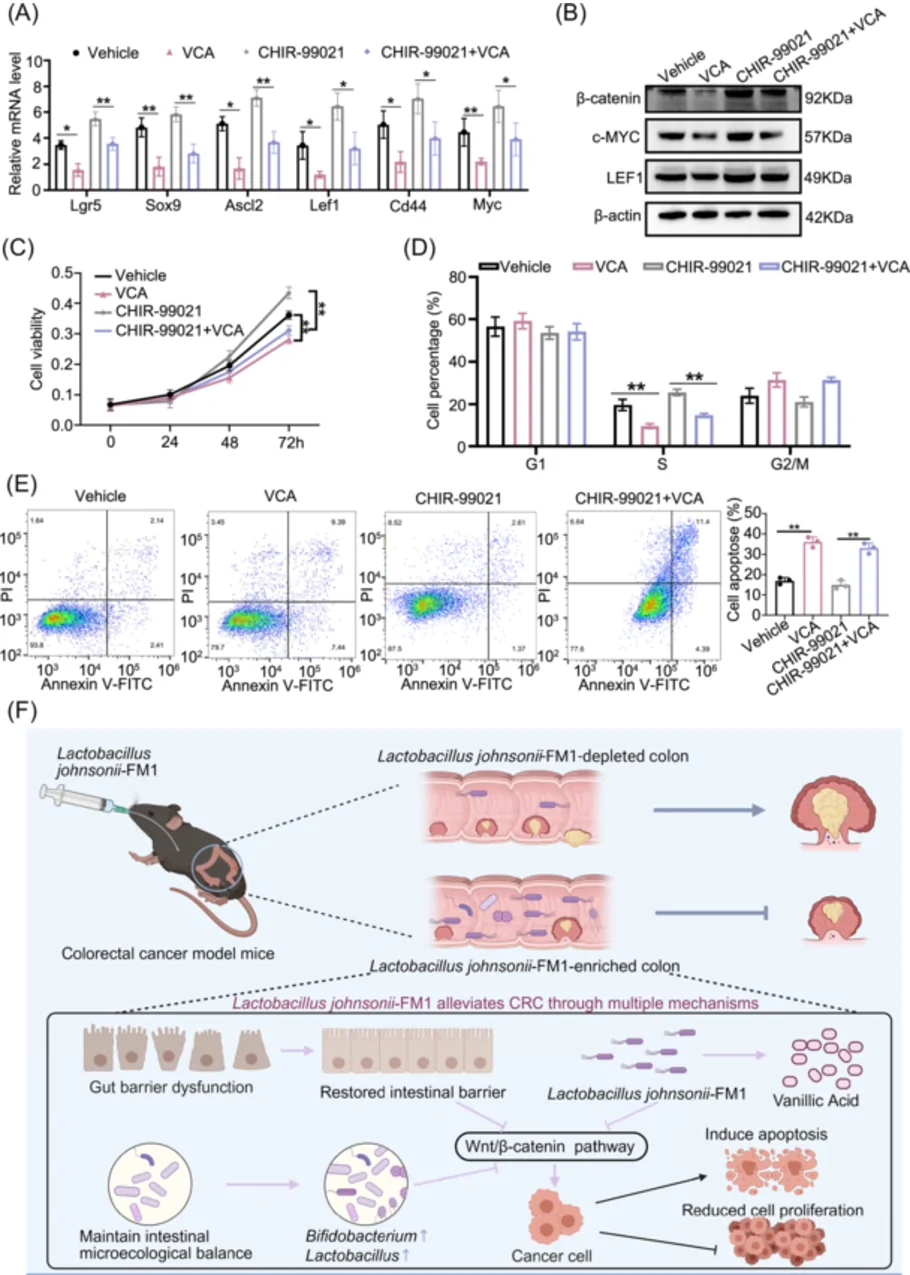
Vanillic acid mediates antitumor activity by suppressing Wnt/β‐catenin signaling. (A) RT‐qPCR analysis of Wnt/β‐catenin target gene mRNA levels (*Lgr5*, *Sox9*, *Ascl2*, *Lef1*, *Cd44*, *Myc*) in HCT116 cells treated with Vehicle, VCA, CHIR‐99021 or CHIR‐99021 + VCA. (B) Western blot of β‐catenin, c‐MYC, and LEF1protein levels under the same treatments. *β*‐actin serves as the loading control. (C) Cell viability assay measured at 0, 24, 48, and 72 h posttreatment with Vehicle, VCA, CHIR‐99021, or CHIR‐99021 + VCA. The *Y*‐axis shows optical density at 570 nm (OD₅₇₀), which corresponds to MTT formazan absorbance and is proportional to the number of viable cells. (D) Flow cytometric analysis of cell‐cycle distribution after treatment with Vehicle, VCA, CHIR‐99021, or CHIR‐99021 + VCA. (E) Annexin V–FITC/PI staining and quantification of apoptotic cells after 48 h treatment with Vehicle, VCA, CHIR‐99021 or CHIR‐99021 + VCA. (F) *Lactobacillus johnsonii*‐FM1 suppresses colorectal tumorigenesis through restoring healthy gut microbiota and metabolites, improving gut barrier function, and subsequently suppressing oncogenic and modulating the Wnt/β‐catenin signaling pathway. Data are expressed as mean ± SD. Statistical significance was determined by one‐way or two‐way analysis of variance, where appropriate. ∗∗*p* < 0.01, ∗*p* < 0.05.

To further validate the involvement of Wnt/β‐catenin signaling in VCA's antitumor activity, we performed rescue experiments in HCT116 using the Wnt/β‐catenin activator CHIR‐99021. Cells were treated with Vehicle, VCA, CHIR‐99021, or CHIR‐99021 pretreatment followed by VCA co‐treatment. We then measured Wnt/β‐catenin target gene expression (β‐catenin, c‐Myc, Cyclin D1) by RT‐qPCR and Western blot, and assessed cell proliferation, cell‐cycle distribution by flow cytometry, and apoptosis. The results showed that CHIR‐99021 alone enhanced Wnt/β‐catenin signaling, as evidenced by a significant upregulation of β‐catenin, c‐Myc, and Cyclin D1 mRNA and protein levels compared with Vehicle. In contrast, VCA alone potently suppressed these targets. Importantly, when VCA was added following CHIR‐99021 pretreatment, it effectively reversed CHIR‐99021‐induced target gene activation, bringing β‐catenin, c‐Myc, and Cyclin D1 expression back down toward baseline (Figure 7A,B). And CHIR‐99021—only treatment increased CRC cell proliferation, reduced the percentage of cells in S‐phase, and decreased apoptotic rates (Figure 7C–E). VCA alone had the opposite effects—dampening proliferation, inducing S‐phase arrest, and elevating both early and late apoptosis. Strikingly, VCA co‐treatment after CHIR‐99021 pretreatment restored cell‐cycle profiles and apoptotic rates to levels comparable to Vehicle (Figure 7C–E). Collectively, these data demonstrated that VCA antagonized CHIR‐99021‐induced Wnt/β‐catenin activation to inhibit proliferation, restore normal cell‐cycle progression, and promote apoptosis, confirming that VCA's antitumor effects are mediated, at least in part, via suppression of the Wnt/β‐catenin pathway.

## 3 DISCUSSION

The widely accepted definition of probiotics is: “live microorganisms that, when administered in sufficient quantities, provide a health benefit to the host [1].” *Lactobacillus johnsonii*, one of the typical gut probiotics, is widely distributed in the gastrointestinal tracts of various hosts, including humans, mice, dogs, poultry, pigs, and bees [35, 36, 37]. *L. johnsonii* plays important roles in several conditions, such as diabetes, respiratory diseases, liver disorders, and intestinal diseases, by exerting anti‐inflammatory effects, modulating the immune system, and maintaining the integrity of the gut barrier [22, 38, 39, 40]. Notably, a recent study reported that stressed colorectal cancer mice exhibited a significant reduction in *L. johnsonii* abundance, which was inversely correlated with tumor burden, further supporting its tumor‐suppressive potential in CRC progression [41]. In our previous research, we observed a significant decrease in *L. johnsonii* abundance in the gut microbiota of various colorectal cancer mouse models, as well as in clinical patients with colorectal cancer [28].

In this study, we used *Lactobacillus johnsonii*‐FM1, a novel strain isolated from the feces of black rice‐fed mice in our previously published study, where a black rice diet was shown to modulate gut microbiota composition and alleviate colorectal cancer progression [27]. We then demonstrated for the first time that oral administration of *L. johnsonii*‐FM1 significantly reduced both the number and size of intestinal tumors in *Apc* ^*Min/+*^ mice. This finding was further confirmed in the AOM/DSS‐induced colorectal cancer mouse model. Additionally, *L. johnsonii*‐FM1 selectively suppressed the viability of CRC cells without affecting normal colonic cells, indicating that *L. johnsonii*‐FM1 inhibits CRC tumorigenesis in mice (Figure 7F).

One of the ways probiotics combat disease is by maintaining gut microbial homeostasis and protecting epithelial barrier function [42]. In this study, we used shotgun metagenomics to analyze changes in mouse gut microbiota following oral administration of *L. johnsonii*‐FM1. We found that *L. johnsonii*‐FM1 significantly increased the abundance of known symbiotic probiotics, including *Bacteroides uniformis* [27], *Bacteroides animalis* [43], and *Parabacteroides distasonis* [44]. *Bacteroides uniformis* can produce the antitumor metabolite indole‐3‐lactic acid, which activates the AHR pathway to inhibit the progression of colorectal tumors [27]. Furthermore, the abundance of potential pathogens such as *Capnocytophaga canimorsus* [45] and *Salmonella enterica* [46] was significantly reduced. Studies have shown that gut dysbiosis increases tumor susceptibility and that alterations in the gut microbiota are key determinants in the development of colorectal tumors [47, 48]. At the same time, some research has found that probiotics can modulate the microbiota composition, thereby alleviating cancer progression. For instance, *Lactobacillus gallinarum* has been shown to inhibit colorectal tumorigenesis by modulating microbial composition [6]. These findings collectively suggest that probiotics like *L. johnsonii*‐FM1 may suppress colorectal cancer development through their ability to modulate the gut microbial composition. Maintaining the balance of gut microbiota can partially sustain intestinal barrier function [49]. In this study, we also assessed the integrity of the intestinal barrier in colorectal cancer mice after oral administration of *Lactobacillus johnsonii*‐FM1. The results revealed that *L. johnsonii*‐FM1 promotes the expression of intestinal barrier proteins ZO‐1 and Occludin, thereby alleviating intestinal damage. Together, this suggests that *L. johnsonii*‐FM1 inhibits CRC, at least in part, by enriching beneficial taxa and reducing potential pathogens, thereby preserving barrier function.

Gut microbiota not only helps counteract colorectal tumor development by maintaining gut microbiota balance but also slows tumor progression through the production of beneficial metabolites, such as short‐chain fatty acids [50, 51]. In this study, we first investigated the effects of LJCS (containing all metabolites) on the growth of various colorectal cancer cell lines and normal epithelial cells. The results showed that the metabolites produced by *L. johnsonii*‐FM1 significantly inhibited the proliferation of colorectal cancer cells and promoted their apoptosis, while having no effect on the proliferation or apoptosis of normal epithelial cells. In other studies, researchers have often focused on identifying key metabolites by differentiating them based on molecular size [6]. However, we believe this approach is insufficient. Therefore, in this study, we utilized metabolomics to further identify the key metabolites produced by *L. johnsonii*‐FM1 that antagonize colorectal cancer. The results showed a significant increase in the abundance of VCA and Gallic acid in the feces of *L. johnsonii*‐FM1‐treated mice. VCA is a natural phenolic compound commonly found in plants and fruits, especially in foods such as blueberries, grapes, and olives [52]. It exhibits various bioactivities, including antioxidant, anti‐inflammatory, and antitumor properties, and studies suggest it may have potential in preventing cancer, cardiovascular diseases, and other metabolic disorders [53, 54]. Although some literature reports VCA as a key metabolite of *L. johnsonii* [41], there is still limited evidence regarding its role in colorectal tumor progression. We explored the effects of VCA both in vivo and in vitro on colorectal cancer cell lines and tumor‐bearing mouse models. The results demonstrated that VCA inhibits colorectal tumor progression by suppressing cell proliferation and promoting apoptosis in cancer cells. Previous studies have highlighted the anticarcinogenic properties of probiotic metabolites, particularly those produced by LAB [55, 56]. For instance, metabolites from *Lactobacillus plantarum* have shown selective cytotoxicity by inducing antiproliferative activity and triggering apoptosis in malignant cancer cells [57]. Thus, the anti‐CRC feature of *L. johnsonii*‐FM1 could also be at least in part attributed to its released protective metabolites.

The adenomatous polyposis coli (APC) gene is a key regulator within the Wnt/β‐catenin signaling pathway, responsible for maintaining the balance between cell proliferation and differentiation [58]. When mutations occur in the APC gene, β‐catenin accumulates in the nucleus, activating the transcription of oncogenes and leading to tumor growth [59, 60]. The Wnt/β‐catenin signaling pathway targets stemness markers such as *Lgr5*, *Sox‐9*, *Axin2*, *Cd44*, *Prom1*, *Oct4*, and *c‐myc* [61]. To further investigate the potential mechanism by which VCA counteracts colorectal tumor progression, we performed transcriptome sequencing on the intestinal tissue of VCA‐treated mice. The results showed a significant downregulation in the expression of genes such as *Lgr5*, *Sox‐9*, *Cd44*, and *c‐myc*. Additionally, a corresponding decrease in the protein levels of these markers was detected in the intestinal tissue. Based on this, we conclude that VCA may inhibit colorectal tumor progression by suppressing the overactivation of the Wnt/β‐catenin signaling pathway. There is still limited research on whether VCA directly regulates the Wnt/β‐catenin signaling pathway. However, studies have shown that VCA can inhibit the cyclic GMP/protein kinase G (cGMP/PKG) signaling pathway, which promotes the proteasomal degradation of β‐catenin [41]. This, in turn, blocks the noncanonical β‐catenin/LEF1/TCF1 axis and counteracts colorectal tumor progression [62].

Although our findings demonstrate that *Lactobacillus johnsonii*‐FM1 exerts significant antitumor effects in a murine model of colorectal cancer, its clinical applicability remains to be determined. This strain was isolated from the feces of black rice‐fed mice, and its ability to colonize the human gut and exert similar therapeutic benefits in humans has not yet been evaluated. Nonetheless, previous studies have reported that other strains of *L. johnsonii*, such as La1 (LC1/NCC 533), are safe for human consumption and have been incorporated into commercial probiotic formulations with documented benefits on gut health and immune modulation [63]. Moreover, the presence of *L. johnsonii*‐based products in international probiotic markets suggests that this species is generally well tolerated and capable of human gut colonization. Given these precedents, *L. johnsonii*‐FM1 holds promise as a candidate for future translational development. However, further studies‐including genomic safety profiling, dose optimization, colonization studies in humanized gut models, and clinical trials‐are essential to fully assess its efficacy and safety in human populations.

In summary, to the best of our knowledge, this is the first study demonstrating the anti‐colorectal cancer effects of *L. johnsonii*‐FM1. *L. johnsonii*‐FM1 has been shown to prevent intestinal tumor formation, an effect associated with its ability to modulate the gut microbiota and secrete protective metabolites, including VCA, which promotes apoptosis in cancer cells. These findings may contribute to the development of probiotic‐based strategies for CRC prevention.

## 4 CONCLUSION

In summary, our study demonstrates that oral administration of *Lactobacillus johnsonii*‐FM1 exerts potent anti‐colorectal cancer effects through multiple, complementary mechanisms. *L. johnsonii* FM1 reconfigures the gut microbial community‐enhancing diversity and enriching commensal taxa—while strengthening intestinal barrier integrity and dampening systemic inflammation. Metabolomic and genomic analyses identify vanillic acid, produced via a conserved VDH pathway, as a key bioactive effector. VCA selectively induces cell‐cycle arrest and apoptosis in colorectal cancer cells and suppresses tumorigenesis in *Apc* ^*Min/+*^ and AOM/DSS mouse models by inhibiting Wnt/β‐catenin signaling. Together, these findings support the therapeutic potential of *L. johnsonii*‐FM1 and its secreted metabolite VCA as a probiotic‐based adjunct strategy for colorectal cancer prevention and treatment.

## 5 MATERIALS AND METHODS

### Animal experiments

Animal experiments were conducted using *Apc* ^*Min/+*^ C57BL/6 mice, a well‐established model for human familial adenomatous polyposis and spontaneous colorectal cancer [64]. At 6 weeks of age, *Apc* ^*Min/+*^ mice were randomly assigned to one of three groups: (1) PBS, (2) *Escherichia coli* MG1655, and (3) *Lactobacillus johnsonii*‐FM1. *L. johnsonii*‐FM1 was cultured in de Man, Rogosa, and Sharpe (MRS) broth (Difco Laboratories), whereas *E. coli* MG1655 was cultured in BHI broth. After 24 h of incubation, bacterial cultures were harvested, resuspended in PBS, and administered to the mice via oral gavage at a dose of 1 × 10^8^ colony‐forming units (CFU) per 100 μL PBS per mouse. The treatment was administered daily for 8 weeks to induce neoplastic lesions. Body weight and stool changes were monitored weekly. VCA ((0.1 mg/mL) catalog number H36001, Sigma) was administered by gavage throughout the experiment, using water as the control. Additionally, the AOM/DSS model was used to simulate human colitis‐associated carcinoma (CAC). C57BL/6 mice at 6 weeks of age received a single intraperitoneal injection of 10 mg/kg AOM (Merck), followed by 2% DSS (MP Biomedicals, Solon) in drinking water for 3 weeks. Post‐induction, AOM/DSS‐treated mice were gavaged with *L. johnsonii*‐FM1 and *E. coli* MG1655 suspensions following the same dosage and schedule as the *Apc* ^*Min/+*^ mice.

Upon the development of neoplastic lesions, mice were anesthetized and euthanized. The small intestines and colons were longitudinally opened, rinsed with PBS, and examined for tumor presence. The total number of tumors in the colon was recorded, and the size of each tumor was measured using a previously published formula [65]. All procedures adhered to the guidelines approved by the Animal Experimentation Ethics Committee of the Huazhong Agricultural University. Ethical approval number: HZAUMO‐2022‐0146.

### Isolation and culture of *L. johnsonii*‐FM1

Sample Collection and Preparation: Fresh fecal pellets (0.1–0.2 g) were collected aseptically from black rice‐fed C57BL/6 mice into sterile 1.5 mL microcentrifuge tubes and immediately placed on ice. Samples were processed within 1 h of collection to minimize microbial shifts. Serial Dilution and Plating: (1) Each fecal sample was suspended in 900 µL sterile phosphate‐buffered saline (PBS; pH 7.4) and vortexed for 1 min to form a homogenous suspension; (2) Serial ten‐fold dilutions were prepared (10^−^¹ through 10^−^⁶) by transferring 100 µL of suspension into 900 µL PBS at each step; (3) From each dilution, 100 µL was spread evenly onto MRS agar plates supplemented with 0.05% l‐cysteine to enhance anaerobic growth; (4) Plates were incubated in an anaerobic chamber (5% H₂, 5% CO₂, 90% N₂) at 37°C for 48–72 h. Preliminary Colony Selection and Morphological Screening: Colonies exhibiting typical *Lactobacillus* morphology (round to slightly irregular, white‐cream, 1–2 mm diameter) were picked and streaked twice on fresh MRS plates to ensure purity. Gram staining was performed on each purified colony to confirm Gram‐positive rod morphology. Molecular Identification: (1) 16S rRNA Gene Sequencing: Genomic DNA was extracted from overnight MRS broth cultures using a commercial kit. The nearly full‐length 16S rRNA gene was amplified with universal primers 27F (5′‐AGAGTTTGATCCTGGCTCAG‐3′) and 1492R (5′‐GGTTACCTTGTTACGACTT‐3′) [66]. Polymerase chain reaction (PCR) products were purified and sequenced (Sanger method). Sequences were compared against EzBioCloud and NCBI databases; isolates with ≥ 99% identity to *L. johnsonii* were retained. (2) Hsp60 Gene Sequencing for Species Discrimination: To distinguish *L. johnsonii* from closely related species (e.g., *L. gasseri*), the heat‐shock protein 60 (*hsp60*) gene was amplified using primers Hsp60‐F (5′‐GAYGAYGCIACIGGITTYGA‐3′) and Hsp60‐R (5′‐CCRTAIACIGCRTGYTCCCA‐3′) [67]. Amplicons were sequenced, and a phylogenetic tree was constructed using MEGA X software (neighbor‐joining method, 1000 bootstrap replicates) to confirm clustering within the *L. johnsonii* clade.

Culture Conditions for Experiments: (1) Routine Maintenance: FM1 was routinely cultured in MRS broth at 37°C under anaerobic conditions with shaking at 120 rpm; overnight cultures reached OD₆₀₀ ≈ 1.8–2.0. (2) Metabolite Production: For VAC extraction, FM1 was grown in BHI broth at 37°C anaerobically for 48 h, as BHI's rich peptide and amino acid content enhances secondary metabolite synthesis. Stock Preparation: Pure FM1 cultures were mixed 1:1 with sterile 40% glycerol and stored at –80°C. Working stocks were revived by streaking onto MRS agar and incubating anaerobically before each experiment.

### RNA sequencing

Total RNA was extracted from cells treated with VCA or PBS using Trizol Reagent (Life Technologies). Three biological replicates were performed for each condition. Sequencing libraries were constructed using the TruSeq Stranded Total Sample Preparation Kit (Illumina). Libraries were sequenced on HiSeq machines based on activity and expected data volume. RNA sequence reads were aligned to the *Homo sapiens* GRCh37/hg19 reference genome. Differential gene expression levels were quantified based on transcript abundance.

### Shotgun metagenomics sequencing and analysis

After ensuring sample quality, we fragmented 500 nanograms of meta‐DNA using a Covaris E220 instrument (Covaris). The fragments were then size‐selected within the 300–700 base pair range through magnetic bead‐based selection. Following this, the DNA segments were repaired and ligated with indexed adapters. The resulting ligation product underwent PCR amplification, followed by hybridization with exon probes and capture on streptavidin beads. The captured DNA was amplified via PCR and converted into a single‐stranded circular (ssCir) library. This ssCir library was subjected to rolling circle amplification to produce DNA nanoballs (DNBs), which were subsequently sequenced on a DNBSEQ platform.

For the fecal metagenomic shotgun sequences, quality filtering was performed using the “trimmomatic‐options” in Kneaddata (v0.10.0) [68], with reads shorter than 50 nucleotides excluded. The filtered reads were aligned to the mouse genome (GRCm39/mm39) using Bowtie2, removing any mouse‐derived DNA. Taxonomic composition of the microbial communities was assessed using Kraken2 (v2.9) and Bracken (v3.0.14). Additionally, pathway and gene family abundance were analyzed with HUMANn3 software (v3.0.1) [68]. Differential bacterial taxa were identified using LEfSe, with the criteria set to LDA > 2 and *p* < 0.05 [69].

### Metabolomics profiling for fecal samples and metabolomics analyses

Fecal samples from each mouse were weighed (50 mg) for metabolomic profiling. Cold methanol (80%) was used for fecal metabolites extraction. The extracted samples were then centrifuged at 21,500 × *g* for 15 min at 4°C, and the supernatant was subjected for LC‐MS/MS analysis. MS raw files were processed using the same method as described above [27].

### Cell viability assay

Cell proliferation was assessed by MTT [3‐(4,5‐dimethylthiazol‐2‐yl)−2,5‐diphenyl tetrazolium bromide] assay (Sigma‐Aldrich). Human colorectal cancer cells (HCT116 or SW620) and normal colonic epithelial cells (NCM460) were seeded at 1 × 10³ cells/well in 96‐well plates and allowed to adhere overnight in complete DMEM (10% FBS, 1% penicillin/streptomycin). Before bacterial treatment, wells were washed once with PBS and medium was replaced with antibiotic‐free DMEM. *L. johnsonii*‐FM1 or *E. coli* cultures were grown to mid‐log phase, washed twice in PBS, and resuspended to 1 × 10⁸ CFU/mL. Bacteria were added at an MOI of 100 (1 × 10⁵ CFU per well) and co‐incubated with cells for 4 h at 37°C under aerobic conditions. After 4 h, wells were washed once with PBS and overlaid with DMEM containing 10% FBS, 1% penicillin/streptomycin, and 50 µg/mL gentamicin for 2 h to kill extracellular bacteria. Cells were then returned to standard culture medium and allowed to recover. MTT assays were performed at 24, 48, and 72 h postinfection: 20 µL of 5 mg/mL MTT solution was added per well and incubated for 4 h. Formazan crystals were solubilized in 150 µL DMSO, and absorbance was measured at 570 nm (reference 630 nm) on a Multiskan GO microplate reader (Thermo Scientific). Under these conditions, OD₅₇₀ values of 0.1–0.2 reflect viable cell numbers of ~2–4 × 10³, consistent with the MOI and 4 h bacterial exposure.

### Statistical analysis

Statistical analyses were performed using GraphPad Prism 8.0 (GraphPad Software Inc.) and R software version 4.0.3. The comparison of categorical variables was conducted using the Chi‐Square Test. For comparison of microbial differences among the three groups (Wild type, CD, and BRD), the Kruskal–Wallis test, a non‐parametric method, was used. If a significant difference was observed, pairwise comparisons between groups were performed using the Mann–Whitney *U* test, and the significance level was adjusted using the Bonferroni correction to control the type I error. Standardized (Z‐score) data were used for metabolite analysis. The covariate effect of species diversity was assessed using multiple linear regression. Intergroup differences for both the Euclidean distance of metabolites and the Bray–Curtis distance of bacteria were tested using PERMANOVA. A significance level of *p* < 0.05 (two‐sided) was applied, and for multiple comparisons, the false discovery rate was controlled using the Benjamini–Hochberg method. Spearman's rank correlation coefficient was used to estimate microbe–microbe or microbe–metabolite correlations. All statistical tests were two‐sided unless otherwise specified.

## AUTHOR CONTRIBUTIONS

**Wei Lyu**: Conceptualization; funding acquisition; writing—original draft; writing—review and editing. **Lu Chen**: Writing—original draft; software; data curation. **De‐Feng Li**: Investigation; methodology. **Shu‐Ying Li**: Validation. **Qian Dai**: Data curation. **Hong‐Li Zhou**: Validation. **Yan‐Yan Liu**: Investigation. **Jian‐Yun Zhou**: Funding acquisition; writing—review and editing; writing—original draft. **Xin‐Jun Liang**: Funding acquisition; writing—original draft; writing—review and editing. **Ling Wang**: Conceptualization; writing—review and editing; writing—original draft; visualization; project administration; formal analysis; supervision; resources.

## CONFLICT OF INTEREST STATEMENT

The authors declare no conflicts of interest.

## ETHICS STATEMENT

All procedures complied with the guidelines were approved by the Animal Experimentation Ethics Committee of Huazhong Agricultural University (No. HZAUMO‐2022‐0146).

## Supporting information

Figure S1: *Lactobacillus johnsonii*‐FM1‐treatment showed no effect on mice body weight, liver, and kidney.
Figure S2: *Lactobacillus johnsonii*‐FM1 protects against gut tumourigenesis in AOM‐DSS‐induced CRC mice.
Figure S3: Effects of *Lactobacillus johnsonii*‐FM1 on gut microbiota at phylum level.
Figure S4: *Lactobacillus johnsonii*‐FM1 altered gut microbial composition and increased the abundance of potential probiotics in the AOM/DSS model mice.
Figure S5: *Lactobacillus johnsonii*‐FM1 restores CRC‐induced gut barrier dysfunction in AOM/DSS mice.
Figure S6: Docking of vanillin to a functionally validated vanillin dehydrogenase (VDH).
Figure S7: Source Distribution and Pathway Enrichment of Co‐Upregulated Metabolites.
Figure S8: VCA inhibits the viability of colon cancer cells.

Table S1: The differential microbes of *L. johnsonii*‐FM1, *E.coil*, and PBS.
Table S2: Differential metabolites in *L. johnsonii*‐FM1 and PBS.
Table S3: Differential metabolites in *L. johnsonii*‐FM1 and *E.coil*.
Table S4: Differential metabolites in *E.coil* and PBS.
Table S5: Candidate Vanillin Dehydrogenase (VDH) Homologs Identified in *L. johnsonii*‐FM1 Genomes.

## Data Availability

The data that support the findings of this study are available from the corresponding author upon reasonable request. The data sets generated in the current study are available in the National Genomics Data Center Beijing Institute of Genomics, Chinese Academy of Sciences/China National Center for Bioinformation (CRA021304) and are publicly accessible at https://ngdc.cncb.ac.cn/gsa/browse/CRA021304. The data and scripts for analysis can be found at https://github.com/lchen1122/LJ_CRC. Supplementary materials (methods, figures, tables, graphical abstract, slides, videos, Chinese translated version, and update materials) may be found in the online DOI or iMeta Science http://www.imeta.science/imetaomics/.
