# Supplementary material for: Lactobacillus johnsonii‐FM1 modulates gut microbiota and secretes anticancer metabolite vanillic acid to inhibit colorectal tumorigenesis
Source: IMetaOmics. 2025 Sep 3;2(3):e70050. doi: 10.1002/imo2.70050 (PMC12806084; doi:10.1002/imo2.70050)
Supplement: Supplementary file 1 — Figure S1: Lactobacillus johnsonii‐FM1‐treatment showed no effect on mice body weight, liver, and kidney. Figure S2: Lactobacillus johnsonii‐FM1 protects against gut tumourigenesis in AOM‐DSS‐induced CRC mice. Figure S3: Effects of Lactobacillus johnsonii‐FM1 on gut microbiota at phylum level. Figure S4: Lactobacillus johnsonii‐FM1 altered gut microbial composition and increased the abundance of potential probiotics in the AOM/DSS model mice. Figure S5: Lactobacillus johnsonii‐FM1 restores CRC‐induced gut barrier dysfunction in AOM/DSS mice. Figure S6: Docking of vanillin to a functionally validated vanillin dehydrogenase (VDH). Figure S7: Source Distribution and Pathway Enrichment of Co‐Upregulated Metabolites. Figure S8: VCA inhibits the viability of colon cancer cells. [file IMO2-2-e70050-s001.docx]

Supporting information to

***Lactobacillus johnsonii-*FM1 Modulates Gut Microbiota and Secretes Anti-Cancer Metabolite Vanillic Acid to Inhibit Colorectal Tumorigenesis**

**Running title:** *Lactobacillus johnsonii-*FM1 Inhibits Colorectal Tumorigenesis via Vanillic Acid

Wei Lyu^1,2^^,3#^, Lu Chen^4#^, De-Feng Li^1^^#^, Shu-Ying Li^1^, Qian Dai^1^, Hong-Li Zhou^1^, Yan-Yan Liu^5^, Jian-Yun Zhou^1^*, Xin-Jun Liang ^6^*, Ling Wang^1,2,3^*

^1^*Clinical Medical Research Center, The Second Affiliated Hospital of Army Military Medical University, Chongqing, 400037, China*

^2^*Department of Pharmaceutical Chemistry, University of California-San Francisco, San Francisco, California, 94158, United States*

^3^*College of Life Science and Technology, College of Biomedicine and Health, Huazhong Agricultural University, Wuhan, 430070, China*

^4^*Institute of Food and Nutrition Development, Ministry of Agriculture and Rural Affairs, Beijing, 100081, China*

*^5^Department of Nephrology, Tongji Hospital of Tongji Medical College, Huazhong University of Science and Technology, Wuhan, 430072, China*

^6^*Department of Medical Oncology, Hubei Cancer Hospital, Tongji Medical College, Huazhong University of Science and Technology, Wuhan, 430070, China*

^#^ These authors contributed equally.

^*^Correspondence: ling.wang@ucsf.edu (Ling Wang), zhoujianyun1983@tmmu.edu.cn (Jian-Yun Zhou), doctorlxj@163.com (Xin-Jun Liang).

**Supporting Materials and methods**

**Cell culture**

Colon cancer cell lines, HCT116 and SW620, were purchased from the American Type Culture Collection (ATCC). A normal colonic epithelial cell line, NCM460, was obtained from INCELL Corporation (San Antonio, TX) to serve as a control. All cell lines were cultured in high-glucose Dulbecco’s Modified Eagle’s Medium (DMEM; Thermo Fisher Scientific) supplemented with 10% fetal bovine serum (FBS; Thermo Fisher Scientific), 50 U/mL penicillin, and 50 μg/mL streptomycin in a humidified atmosphere containing 5% CO_2_.

**Serum LPS,** **TNF-α, IL-4, IL-6, and IL-10 quantification**

Serum levels of LPS, TNF-α, IL-4, IL-6, and IL-10 were assessed using ELISA kits (LPS: catalog number RK04263, ABclonal, Wuhan, China; TNF-α: catalog number RK00027, ABclonal, Wuhan, China; IL-4: catalog number RK00036, ABclonal, Wuhan, China; IL-6: catalog number RK00008, ABclonal, Wuhan, China; IL-10: catalog number RK00016, ABclonal, Wuhan, China). All experimental procedures were conducted in accordance with the manufacturer's instructions.

**Immunohistochemistry staining**

Paraffin-embedded intestinal tissue cut into 4 μm slices by a microtome were also subjected to immunostaining for detecting the expressions of Ki-67, Claudin-3, and Occludin with primary antibodies of Ki-67 (catalog number 16667; Abcam, Cambridge, MA), ZO-1 (catalog number 33-9100; Thermo Fisher Scientific, Waltham, MA), Claudin-3 (catalog number 34-1700; Thermo Fisher Scientific, Waltham, MA), and Occludin (catalog number PA5-30230; Thermo Fisher Scientific, Waltham, MA), and 6 areas randomly selected from each section were viewed at the tumor tissue. The percentage of positive cells in each field was calculated by Image J.

**Culture supernatant of *L. johnsonii***

After culturing *L. johnsonii* or *Escherichia coli* MG1655 in brain heart infusion (BHI) broth for 1-2 days, the bacterial concentration in each culture medium was measured using a NanoDrop spectrophotometer (NanoDrop Technologies, Wilmington, DE). The BHI cultures were then diluted to ensure equal concentrations of *L. johnsonii* and *E. coli*. Culture supernatants were collected by centrifugation at 5,500 rpm for 15 minutes, followed by sterile filtration using a 0.22-μm membrane. The filtrates were termed *L. johnsonii* culture supernatant (LJCS) or *E. coli* MG1655 culture supernatant (ECCS). The bacterial supernatants were subsequently subjected to heat treatment at 100°C for 30 minutes or treated with proteinase K (50 µg/mL; QIAGEN GmbH, Hilden, Germany). The treated supernatants were used at a concentration of 10% (v/v) for the 3-(4,5-dimethylthiazol-2-yl)-2,5-diphenyltetrazolium bromide (MTT) assay.

**Transmission electron microscopy**

Colon tissue samples were fixed in 2.0% glutaraldehyde in 0.1 mol/L sodium cacodylate (Electron Microscopy Sciences, Hatfield, PA). The fixed tissues were then sectioned into ultrathin slices using a Reichert Ultracut E ultramicrotome. The ultrastructure of the tissue samples was examined using a Philips CM100 transmission electron microscope.

**Colony Formation Assay**

Colon cells (1,000 per well) were seeded onto 6-well plates and treated with St.CM (1% St.CM in DMEM). BHI and Escherichia coli CM served as controls. The treatment medium was replaced every 3 days. After culturing for 14 to 18 days, cells were fixed with 70% ethanol and stained with a 0.5% crystal violet solution. Colonies containing more than 50 cells were counted. All experiments were conducted in triplicate, with each experiment performed three times.

**EdU Cell Proliferation Assay**

Cells were incubated with EdU (10 µM final concentration) for 2 hours prior to fixation. After washing with PBS, cells were fixed with 4% paraformaldehyde and permeabilized with 0.5% Triton X-100. The incorporated EdU was detected using a Click-iT™ EdU Imaging Kit (catalog number C10338; Thermo Fisher Scientific, Waltham, MA Thermo Fisher,) according to the manufacturer’s instructions. Nuclei were counterstained with Hoechst 33342, and EdU-positive cells were visualized using a fluorescence microscope. The proliferation index was calculated as the percentage of EdU-positive cells relative to total nuclei.

**Cell cycle progression**

To assess cell cycle progression, cells underwent a 24-hour serum starvation, followed by treatment in DMEM containing either 0.1% fatty acid-free bovine serum albumin (BSA)/BHI, ECCS, LJCS, VCA and PBS in 0.1% fatty acid-free BSA for 12 hours. Subsequently, cells were fixed in 70% ethanol and stained with propidium iodide for DNA content analysis. Flow cytometry was employed to evaluate the distribution of cells across distinct cell cycle phases.

**Flow Cytometry**

For cell cycle analysis, treated cells were fixed with 70% ethanol overnight. They were then stained with 50 μg/mL propidium iodide (PI) (BD Pharmingen, San Jose, CA) for 30 minutes at 4°C in the dark. Ten thousand cells were analyzed using a FACSAria cell sorter (BD Biosciences, Franklin Lakes, NJ), and cell cycle profiles were determined using ModFit 3.0 software (Verity Software House, Topsham, ME). Apoptotic cells were assessed using the annexin V apoptosis assay. Treated cells were collected and resuspended in 100 μL annexin-binding buffer (10 mmol/L HEPES, 140 mmol/L NaCl, 2.5 mmol/L CaCl2, pH 7.4) containing 5 μL of annexin V conjugated with allophycocyanin and 50 μg/mL PI. After a 15-minute incubation at room temperature, cells were mixed with an additional 400 μL of ice-cold annexin-binding buffer and analyzed using the FACSAria cell sorter.

**Supporting Figure**

**
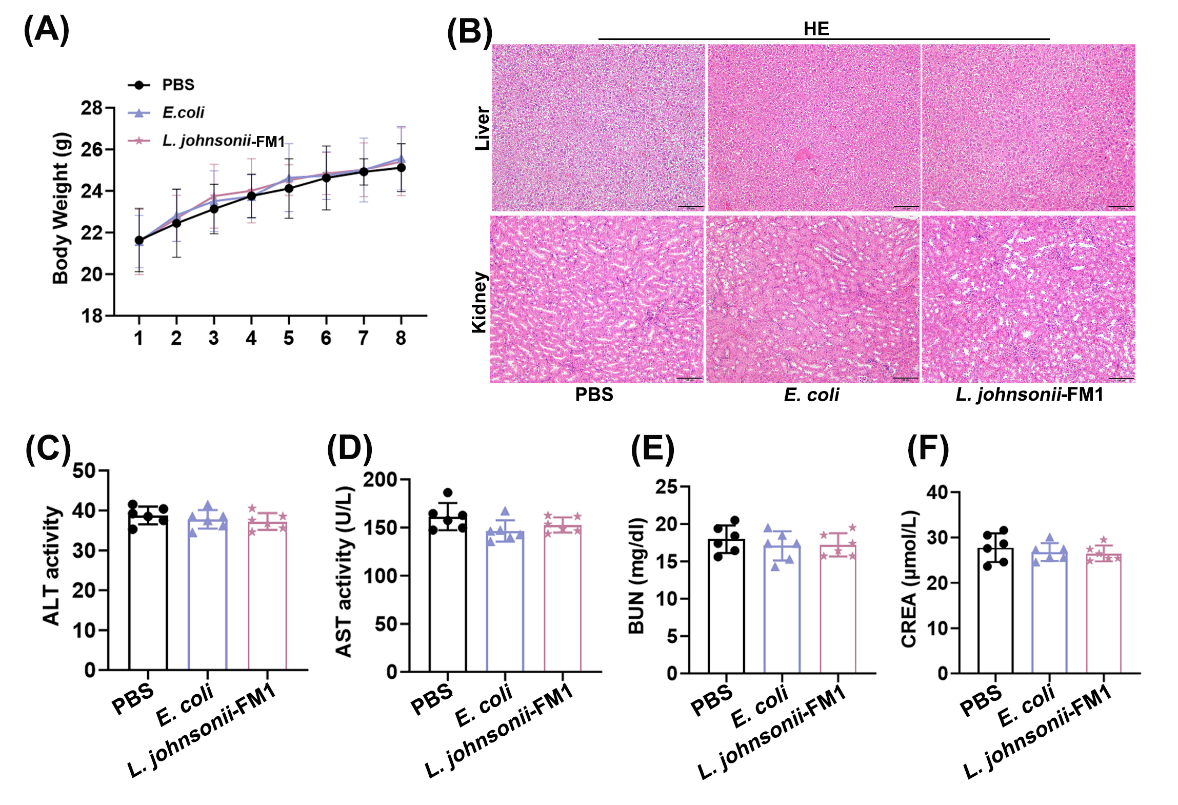
**

**Figure S1: *Lactobacillus johnsonii*-FM1-treatment showed no effect on mice body weight, liver and kidney.** (A) Mice body weight of *Apc^Min/+^* mice treat with PBS, *E. coli* and *L. johnsonii*-FM1. (B) H&E staining was used for pathological evaluation of the mouse liver and kidney. (C-F) Serum ALT and AST level and serum Blood Urea Nitrogen (BUN) and Creatinine (CREA) level, of *Apc*^Min/+^ CRC mouse models. Data are expressed as mean ± Standard Deviation (SD). Dot plots reflect data points from independent experiments.

**
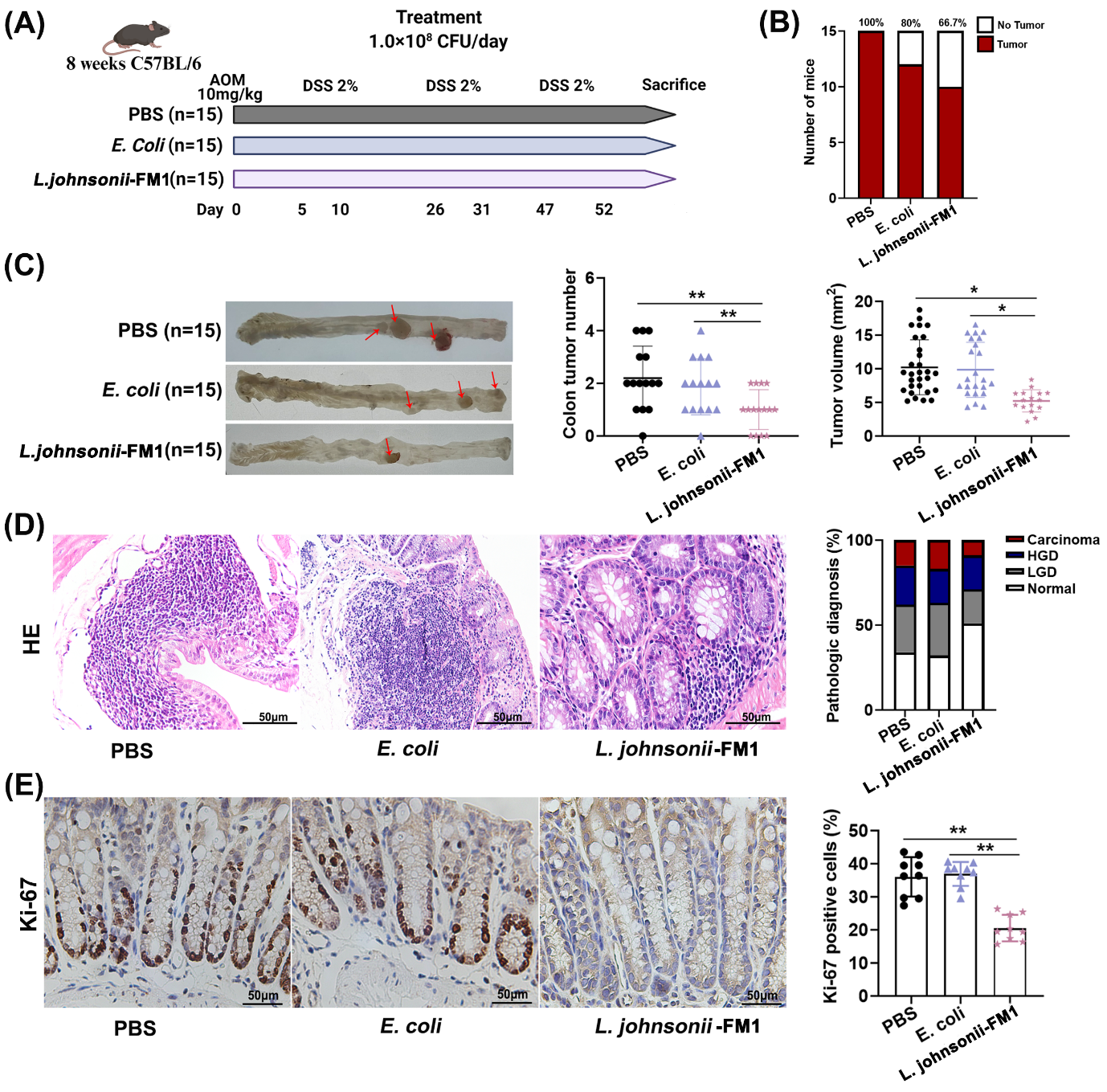
Figure S2: *Lactobacillus johnsonii*-FM1 protects against gut tumourigenesis in AOM-DSS-induced CRC mice.** (A) Schematic diagram showing the experimental design, timeline of AOM/DSS mouse model. (B) The number of mice with hematochezia events in different groups was counted, and the proportion of all mice in the group was calculated (n=15). (C) Representative images of colon tumors from AOM/DSS mouse model. Colon tumor number and tumor size in AOM/DSS mice under different treatments. (D) H&E staining for pathologic diagnosis of mice colons. Quantitative analysis of pathologic score was calculated according to the following criteria: 0 for normal, 1 for low-grade [dysplasia](https://www.sciencedirect.com/topics/medicine-and-dentistry/dysplasia) (LGD), 2 for high-grade [dysplasia](https://www.sciencedirect.com/topics/pharmacology-toxicology-and-pharmaceutical-science/dysplasia) (HGD), and 3 for [carcinoma](https://www.sciencedirect.com/topics/pharmacology-toxicology-and-pharmaceutical-science/carcinoma). (E) The immunohistochemical (IHC) staining for Ki-67 of mice colons. IHC staining for Ki-67 of mice colons with quantitative analysis of Ki-67 index. AOM, azoxymethane; DSS, dextransulfatesodium. Data are expressed as mean ± SD. Statistical significance was determined by 1-way or 2-way analysis of variance, where appropriate. ^∗∗^*P*<0*.*01, ^∗^*P*<0*.*05. Dot plots reflect data points from independent experiments.


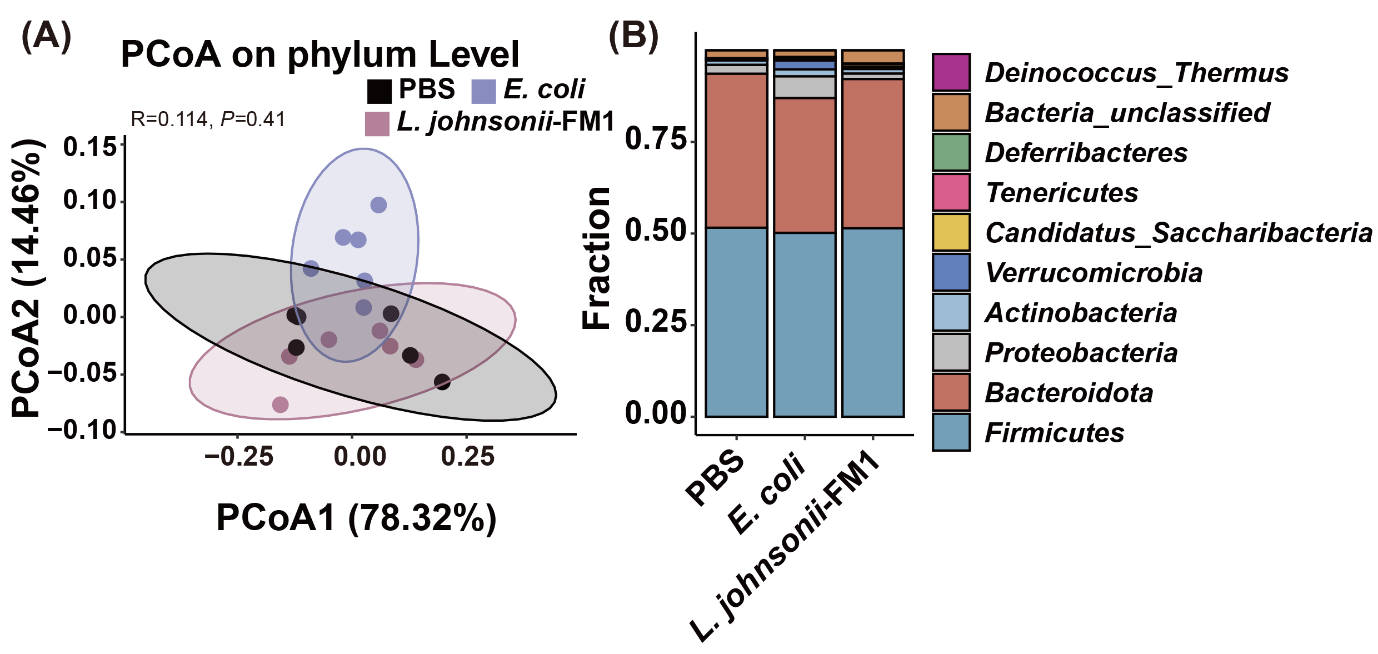


**Figure S3: Effects of *Lactobacillus johnsonii*-FM1 on gut microbiota at phylum level.** (A) Principal Coordinate Analysis 2 (PCoA2 analysis) (β-diversity) on phylum level of the gut microbiota in control and *L. johnsonii*-FM1-treated mice. (B) Identification of marker microbes differentiating groups. Dot plots reflect data points from independent experiments.

**
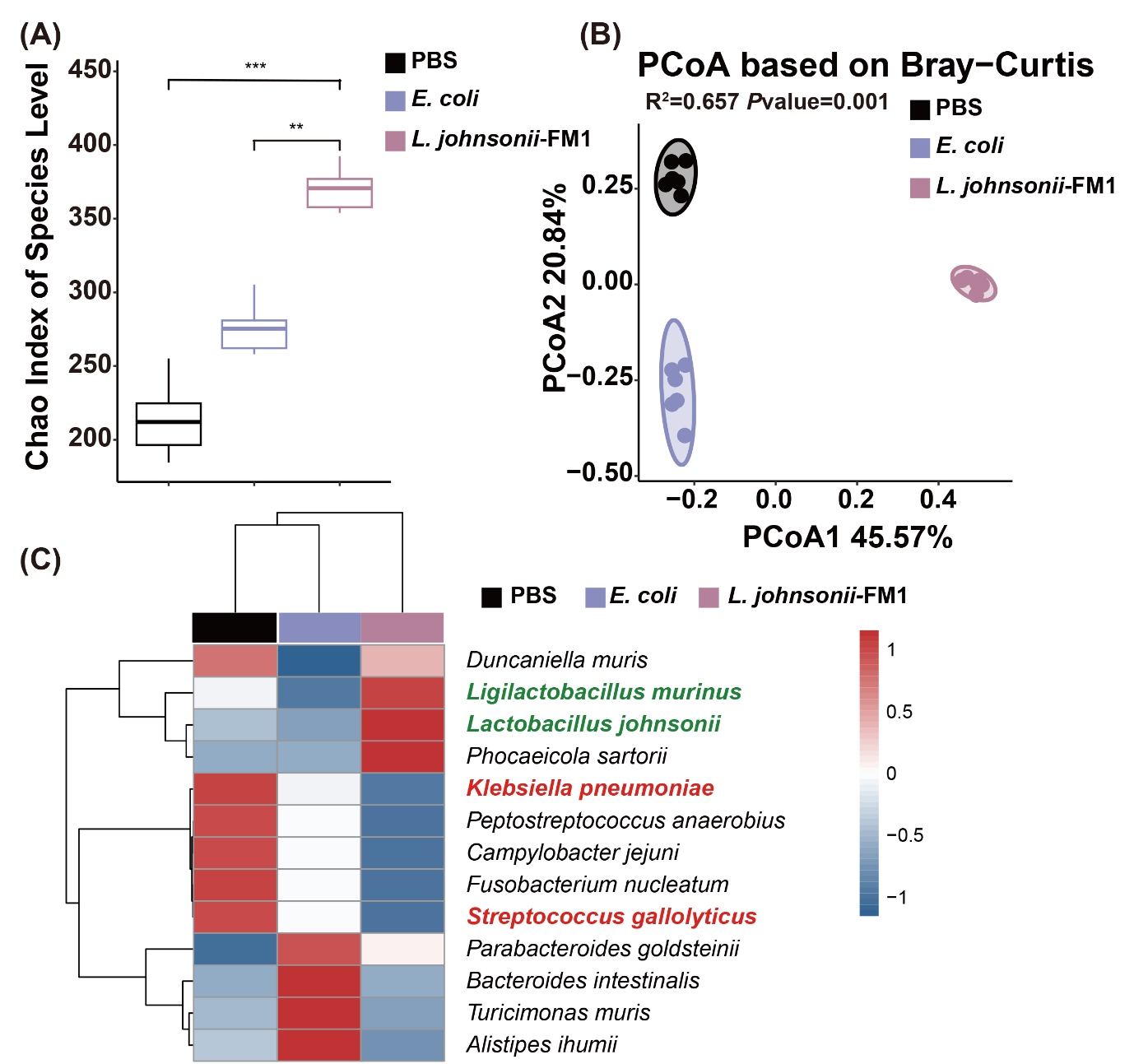
**

**Figure S4: *Lactobacillus johnsonii*-FM1 altered gut microbial composition and increased the abundance of potential probiotics in the AOM/DSS model mice.** (A-B) Chao Index (α-diversity) and PCoA2 analysis (β-diversity) of the gut microbiota in control and *L. johnsonii*-FM1-treated mice from the AOM/DSS model. Comparisons of α- and β-diversity were accessed by 2-tailed Mann-Whitney U test and permutational multivariate analysis of variance, respectively. (C) Identification of marker microbes differentiating groups between *L. johnsonii*-FM1 and *E. coli* or between the PBS (*P* < 0.05, LDA > 2).

**
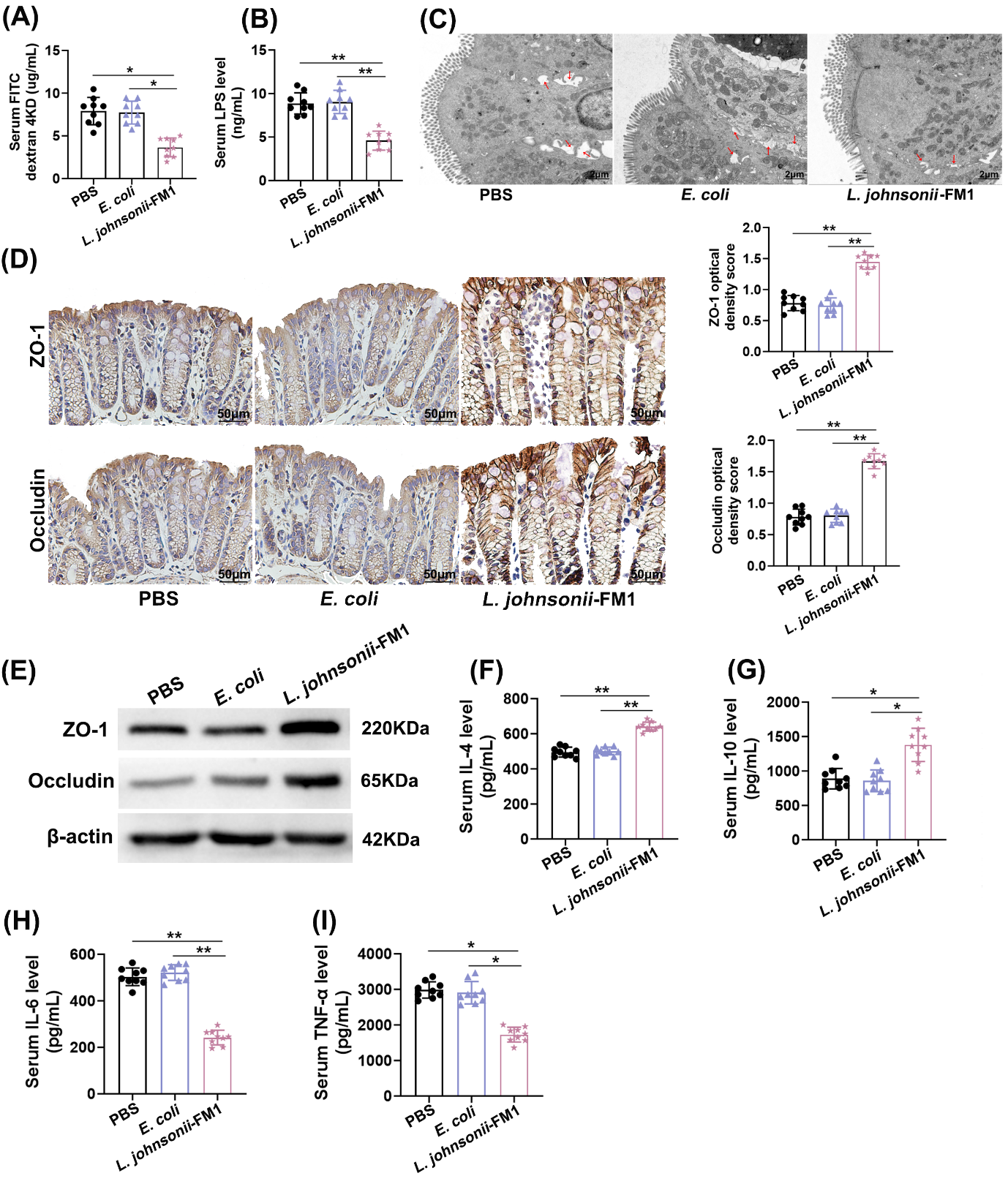
Figure S5: *Lactobacillus johnsonii*-FM1 restores CRC-induced gut barrier dysfunction in AOM/DSS mice.** (A-B) Serum FITC-dextran 4 KD concentration and lipopolysaccharide (LPS) concentration of control and *L. johnsonii*-FM1-treated mice from AOM/DSS model (n=9). (C) Representative images of [intercellular junctions](https://www.sciencedirect.com/topics/medicine-and-dentistry/cell-junction) of control and *L. johnsonii*-FM1-treated mice by [transmission electron microscopy](https://www.sciencedirect.com/topics/pharmacology-toxicology-and-pharmaceutical-science/transmission-electron-microscopy) (TEM). (D) Immunohistochemical (IHC) for distribution of the [adhesion molecule](https://www.sciencedirect.com/topics/medicine-and-dentistry/cell-adhesion-molecule) ZO-1 and Occludin with quantitative analysis in colon tissues of PBS, *Ecoli* and *L. johnsonii*-FM1-treated mice from AOM/DSS model. (E) Expression of gut barrier-associated proteins ZO-1 and Occludin in colon tissues of PBS, *Ecoli* and *L. johnsonii*-FM1-treated mice from AOM/DSS model by [Western blot](https://www.sciencedirect.com/topics/medicine-and-dentistry/western-blot). (F-G) Anti‐inflammatory interleukin (IL‐4) and IL‐10 concentrations and (H-I) pro‐inflammatory IL‐6 and TNF‐α concentrations in serum of *L. johnsonii*-FM1-treated group and control group in an AOM/DSS model. Data are expressed as mean ± SD. Statistical significance was determined by 1-way or 2-way analysis of variance, where appropriate. ^∗∗^*P*<0*.*01, ^∗^*P*<0*.*05. Dot plots reflect data points from independent experiments.

**
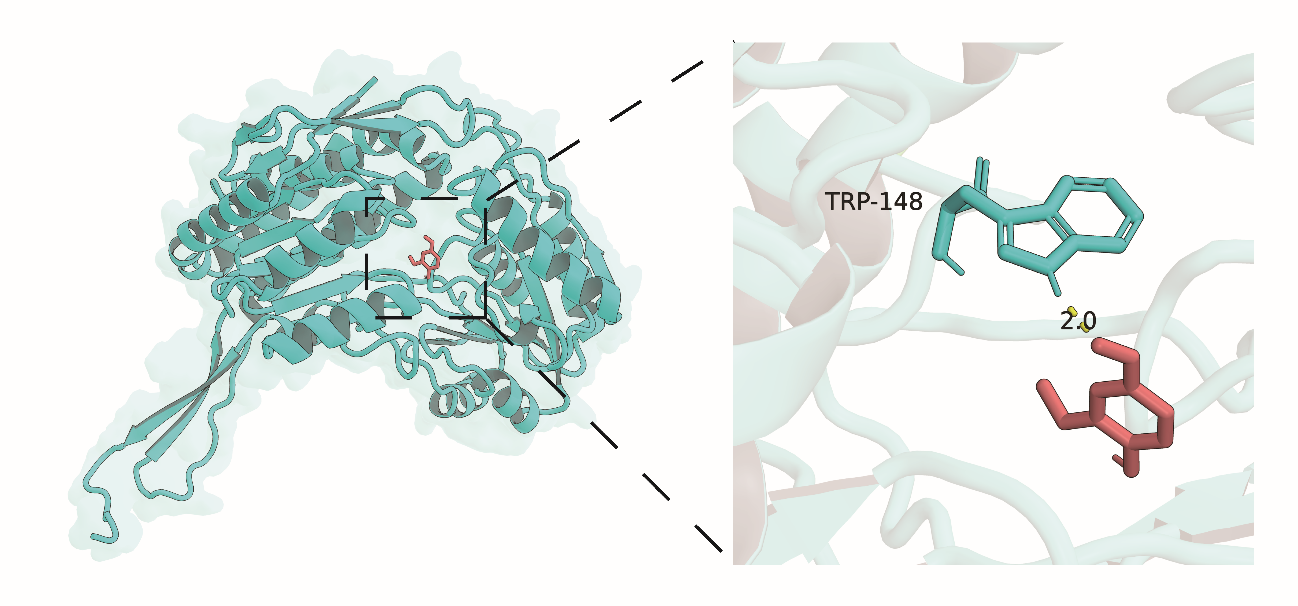
**

**Figure S6:** Docking of vanillin to a functionally validated vanillin dehydrogenase (VDH). Vanillin (red) binds within the catalytic site of the reference VDH enzyme, forming a hydrogen bond with Trp148 at 2.0 Å. The conserved interaction geometry and residue positioning support a similar recognition mode as observed in the FM1-derived protein.

**
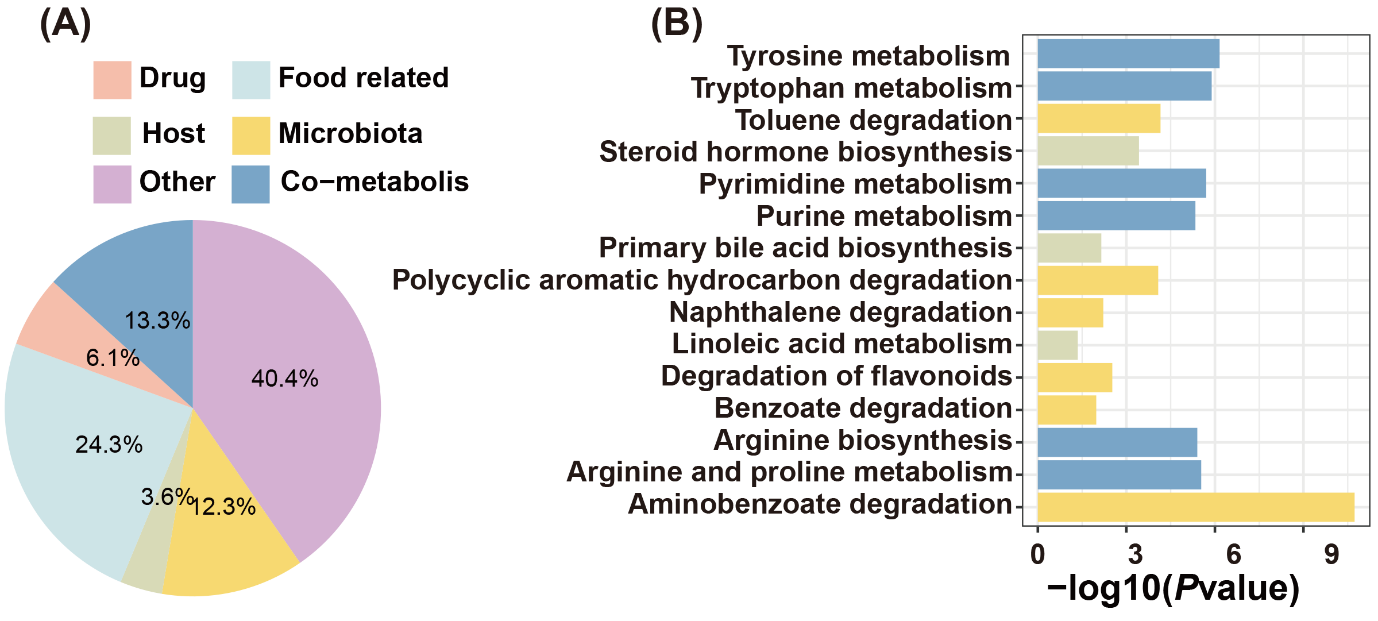
**

**Figure S7: Source Distribution and Pathway Enrichment of Co-Upregulated Metabolites.** (A) Analysis of differential metabolite traceability: percentage of host (3.6%), microbial (12.3%), shared (13.3%), food‐related (24.3%), and other (40.4%) sources. (B) Enrichment analysis of the co‐upregulated metabolites from *L. johnsonii*-FM1-treated *Apc^Min^*^/+^ mice.

**
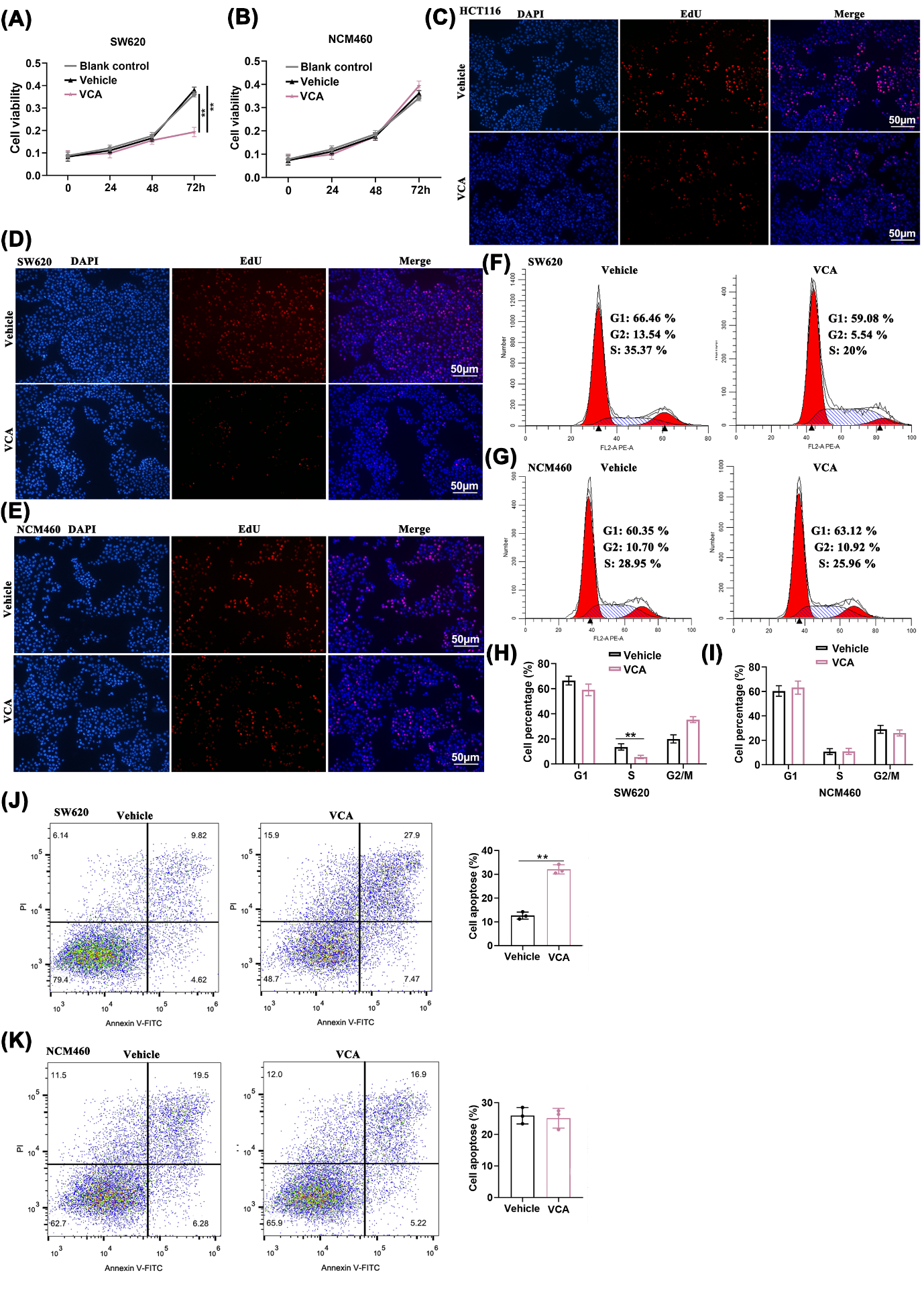
Figure S8: VCA inhibits the viability of colon cancer cells.** (A-B) Vanillic Acid (VCA) reduced the colonic cell viability in SW620, except NCM460. The Y-axis shows optical density at 570 nm (OD₅₇₀), which corresponds to MTT formazan absorbance and is proportional to the number of viable cells. (C-E) VCA reduced EDU-positive CRC cell. (F-I) VCA induced CRC cell cycle arrest at growth (G)_1_/Synthesis (S) phase in SW620 cell line. (J-K) VCA increased CRC cell apoptosis except NCM460. Data are expressed as mean ± SD. Statistical significance was determined by 1-way or 2-way analysis of variance, where appropriate. ^∗∗^*P*<0*.*01, ^∗^*P*<0*.*05. Dot plots reflect data points from independent experiments.
